# Unclear Insomnia Concept in Randomized Controlled Trials and Systematic Reviews: A Meta-Epidemiological Study

**DOI:** 10.3390/ijerph191912261

**Published:** 2022-09-27

**Authors:** Masahiro Banno, Yasushi Tsujimoto, Kunihiro Kohmura, Eisuke Dohi, Shunsuke Taito, Hidehiro Someko, Yuki Kataoka

**Affiliations:** 1 Department of Psychiatry, Seichiryo Hospital, Tsurumai 4-16-27, Showa-ku, Nagoya 466-0064, Japan; 2 Department of Psychiatry, Nagoya University Graduate School of Medicine, Tsurumai-cho 65, Showa-ku, Nagoya 466-8560, Japan; 3 Scientific Research WorkS Peer Support Group (SRWS-PSG), Koraibashi, Chuo-ku, Osaka 541-0043, Japan; 4 Department of Health Promotion and Human Behavior, Kyoto University Graduate School of Medicine/School of Public Health, Kyoto University, Yoshida Konoe-cho, Sakyo-ku, Kyoto 606-8501, Japan; 5 Oku Medical Clinic, Shimmori 7-1-4, Asahi-ku, Osaka 535-0022, Japan; 6 Department of Mental Disorder Research, National Institute of Neuroscience, National Center of Neurology and Psychiatry, Ogawahigashi-cho 4-1-1, Kodaira 187-8502, Japan; 7 Division of Rehabilitation, Department of Clinical Practice and Support, Hiroshima University Hospital, 1-2-3, Kasumi, Minami-ku, Hiroshima 734-8551, Japan; 8 Department of General Internal Medicine, Asahi General Hospital, I 1326, Asahi 289-2511, Japan; 9 Department of Internal Medicine, Kyoto Min-iren Asukai Hospital, Tanakaasukaicyo 89, Sakyo-ku, Kyoto 606-8226, Japan; 10 Section of Clinical Epidemiology, Department of Community Medicine, Kyoto University Graduate School of Medicine, Shogoin Kawara-cho 54, Sakyo-ku, Kyoto 606-8501, Japan; 11 Department of Healthcare Epidemiology, Kyoto University Graduate School of Medicine/School of Public Health, Yoshida Konoe-cho, Sakyo-ku, Kyoto 606-8501, Japan

**Keywords:** insomnia, randomized controlled trials, systematic reviews, meta-epidemiological study

## Abstract

There are two possible ways to conceptualize the term “insomnia”: insomnia disorder and insomnia symptoms, which are often poorly reported. The purpose of this study was to examine the proportion of randomized controlled trials (RCTs) and systematic reviews (SRs) that mention insomnia in their abstracts and cannot distinguish between insomnia disorder and insomnia symptoms from the abstract. We included RCT and SR articles that included the word “insomnia” in the methods or results sections of their structured abstracts, published after 2010. We searched PubMed using English language restrictions on 10 March 2022. From 1580 PubMed articles, we obtained 100 random samples each for eligible RCTs and SRs. The unclear insomnia concept accounted for 88% of the RCT abstracts and 94% of the SR abstracts. Among the RCT and SR abstracts with unclearness, the concept of insomnia was unclear in 27% of RCTs and 57% of SRs after investigating the full text. The concept of insomnia has been unclear in many RCTs and SRs abstracts. The authors of RCTs and SRs are recommended to state “insomnia disorder” or “insomnia symptoms” in the methods and results sections of their abstracts.

## 1. Introduction

The poor reporting quality of randomized controlled trials (RCTs) and systematic reviews (SRs) has caused many problems. For example, poor reporting of treatment in 36% (20/55) of RCTs and in 88% (22/25) of SRs, published in *Evidence Based Medicine* from October 2005 to October 2006, made it difficult to compare the results of these studies with those of other studies [1,2]. Moreover, poor reporting of the results of primary outcomes and adverse events in RTCs and SRs might hinder the application of the article’s results to patient treatments [3,4].

In the field of sleep medicine, the concept of insomnia as a treatment target and outcome has not been properly reported [5,6]. Insomnia may refer to insomnia disorder or insomnia symptoms, and the two definitions are not interchangeable. Insomnia disorder is operationally diagnosed when both nocturnal insomnia symptoms and daytime dysfunctions continue for at least 1 month according to the tenth revision of the International Statistical Classification of Diseases and Related Health Problems (ICD-10), and for at least 3 months according to the third edition of the International Classification of Sleep Disorders (ICSD-3) and the Diagnostic and Statistical Manual of Mental Disorders, Fifth Edition (DSM-5) [7,8,9,10]. The prevalence of insomnia disorder ranges from 4.7% to 22.1% in the cross-cultural general population [11]. The broad concept of insomnia symptoms includes insomnia disorder. The operationalized criteria of nocturnal insomnia symptoms are often used, for example, “taking >30 min to fall asleep, spending >30 min awake after sleep onset, or awakening >30 min before the desired time and before obtaining 6.5 h of sleep” [7]. Nocturnal insomnia symptoms include difficulties initiating sleeping in bed, frequent or continued waking up, or early morning waking up [7]. The prevalence of insomnia symptoms worldwide is 30–35% [11]. Therefore, insomnia disorder and insomnia symptoms are two different concepts, however, they are often confusingly referred to as “patients with insomnia”. Indeed, there are several RCT and SR abstracts in which readers cannot determine whether participants had insomnia disorder or insomnia symptoms (insomnia concept) [5,6,12,13,14,15,16,17,18]. When the study description provided in the abstract is unclear, the abstract results may be misinterpreted. Clinicians and researchers face an increased burden of reading such papers to apply them in clinical practice. Those who do not specialize in sleep medicine may misunderstand the content of the paper.

The proportion and characteristics of RCTs and SRs with unclear definitions of insomnia in their abstracts are unknown. To clarify this point, we investigated the following three questions. First, what is the proportion of RCT and SR articles that did not distinguish between insomnia disorder and insomnia symptoms in their methods or results of their abstracts? Second, among RCT and SR abstracts with unclear definitions, what would be the percentage of the unclear insomnia concept if we consider what is stated in the full text? Third, what are the characteristics of the unclearness of abstracts and the relationship between the characteristics and unclearness of abstracts?

## 2. Materials and Methods

### 2.1. Study Design

This meta-epidemiological study investigated RCT and SR articles published in PubMed. We registered the protocol for this study in protocols.io [19]. We partially adhered to the Preferred Reporting Items for Systematic Reviews and Meta-Analyses (PRISMA) 2020 guidelines, as we performed a systematic search (Table S1) [20].

### 2.2. Types of Studies Included

The inclusion criteria for RCTs and SRs were as follows: (1) RCTs and SRs with structured abstracts; (2) articles included the word “insomnia” in their methods, results, or similar parts of their abstracts. We included abbreviations which contained the word “insomnia” listed in the methods or results, such as cognitive behavioral therapy for insomnia (CBT-I). Conference abstracts and protocols were excluded from this study. We limited the articles to those written in English and those published from 1 January 2010 to 10 March 2022.

### 2.3. Search Methods

We searched MEDLINE via PubMed, with English language restrictions, on 10 March 2022. The search strategy for RCTs was insomnia[tiab] AND method*[tiab] AND randomized controlled trial [pt] AND English [Filter] AND 2010/1/1:3000/12/12[PDAT]. The search strategy for SRs was insomnia[tiab] AND method*[tiab] AND systematic [sb] AND English [Filter] AND 2010/1/1:3000/12/12[pdat]. We used “method*[tiab]” in the search strategy to exclude unstructured abstracts.

### 2.4. Study Selection and Data Extraction

We performed random sampling of 100 RCTs and 100 SRs for statistical analyses of the search results for RCTs and SRs, following the methods of previous studies [21]. Random sampling was continued until the goal of 100 articles per study type was reached. The articles were randomly sorted and screened until the target sample size was reached. Five authors working in pairs (Masahiro Banno (M.B.), Kunihiro Kohmura (K.K.), Eisuke Dohi (E.D.), Shunsuke Taito (S.T.), and Hidehiro Someko (H.S.)) independently reviewed the eligibility criteria for titles and abstracts. M.B. obtained the full text of the included RCTs and SRs and excluded conference abstracts. Then, five authors working in pairs (M.B., K.K., E.D., S.T., and H.S.) independently reviewed the full text according to the eligibility criteria. Disagreements were resolved by discussion. If necessary, other review authors arbitrated the disagreements.

Two of the five authors (M.B., K.K., E.D., S.T., and H.S.) independently extracted information about whether the RCT and SR abstracts described the insomnia concept clearly and the following characteristics: year of publication, impact factor, word count, word limitation in abstracts, funding, compliance with reporting guidelines, registrations or protocols, and journal names that included sleep. Disagreements were resolved by discussion. If necessary, other review authors arbitrated the disagreements. Yuki Kataoka (Y.K.) executed web scraping from the Web of Science with Python 3.6 (Python Software Foundation) and collected data on impact factor and funding on 7 June 2022. We extracted the year of publication (years of print and electronic publication), word counts in abstracts, funding, compliance with reporting guidelines, registrations or protocols, journal names that included sleep from the articles in PubMed, and the full text of each set of RCT and SR articles.

### 2.5. Statistical Analysis

We assigned ‘‘clear insomnia concept’’ to RCT and SR articles that mentioned insomnia concept in the methods or results of their abstracts and assigned the “unclear insomnia concept” to those that did not. We considered the “clear insomnia concept” if the methods or results of their abstracts included the phrase “insomnia disorder”, “primary insomnia”, “secondary insomnia”, or “insomnia symptoms”. We categorized primary and secondary insomnia as “insomnia disorder”. The definitions of the other variables and extraction methods are described in Table S2. The cutoff values for categorization of the continuous variables, impact factors, and word limitation in abstracts were determined based on previous studies [22,23].

We calculated the proportions of RCT and SR abstracts that had an the “unclear insomnia concept”. We investigated tendencies in RCT and SR abstracts for the “unclear insomnia concept” using the calendar year, by applying the Cochran–Armitage test. We also calculated the proportion of included articles with abstract word counts below the upper limit in the instructions for the authors.

Among the RCT and SR abstracts with the unclear insomnia concept, we tabulated the percentages of the unclear insomnia concept based on the description of the full text. We determined full texts with the “unclear insomnia concept” if they lacked the definition of insomnia or non-standard criteria for insomnia (for example, objective total sleep time) were used. We considered that articles mentioned “insomnia disorder” if the standard criteria, such as Diagnostic and Statistical Manual of Mental Disorders (DSM), International Statistical Classification of Diseases and Related Health Problems (ICD), and International Classification of Sleep Disorders (ICSD) for insomnia disorder (including primary insomnia) were used for diagnosis of insomnia disorder or if both subjective insomnia symptoms and daytime dysfunctions continued for one month or more. We considered that RCT and SR articles mentioned “insomnia symptoms” if insomnia was evaluated by a patient-reported sleep diary and patient-reported rating scales (for example, Insomnia Severity Index (ISI), and Pittsburgh Sleep Quality Index (PSQI)) [7]. Among the RCT and SR abstracts with the unclear insomnia concept and with the intention of insomnia disorder based on the description of the full text, we tabulated the percentages of the unclearness about the diagnostic criteria used and who performed the diagnosis, based on the full text.

We also compared the characteristics between RCT and SR abstracts with the “unclear insomnia concept” and those with the “clear insomnia concept” using Fisher’s exact test for categorical variables and the Mann–Whitney U test for continuous variables. We investigated the above possible predictors associated with the unclear insomnia concept using multivariable logistic regression. We calculated crude and adjusted odds ratios using logistic models by penalized maximum likelihood regression to solve the problem of separation in logistic regression [24,25].

Statistical significance was defined as a two-sided *p*-value less than 0.05. We did not adjust the alpha level for multiple comparisons because this was an exploratory study [26]. We used Stata ver. 15.1 (StataCorp LLC, College Station, Texas, United States) for all statistical analyses [27].

### 2.6. Differences between the Protocol and the Article

We calculated the proportion of included articles with abstract word counts below the upper limit in the instructions for the authors as a post hoc analysis. We added journal names that included sleep, as a characteristic of the included articles. Because of the small number of events, we did not include the variable ”year of publication” in the multivariable logistic regression.

### 2.7. Ethics

Ethics approval was not essential because we only used openly available data.

## 3. Results

### 3.1. Selection Process

**Figure 1:**
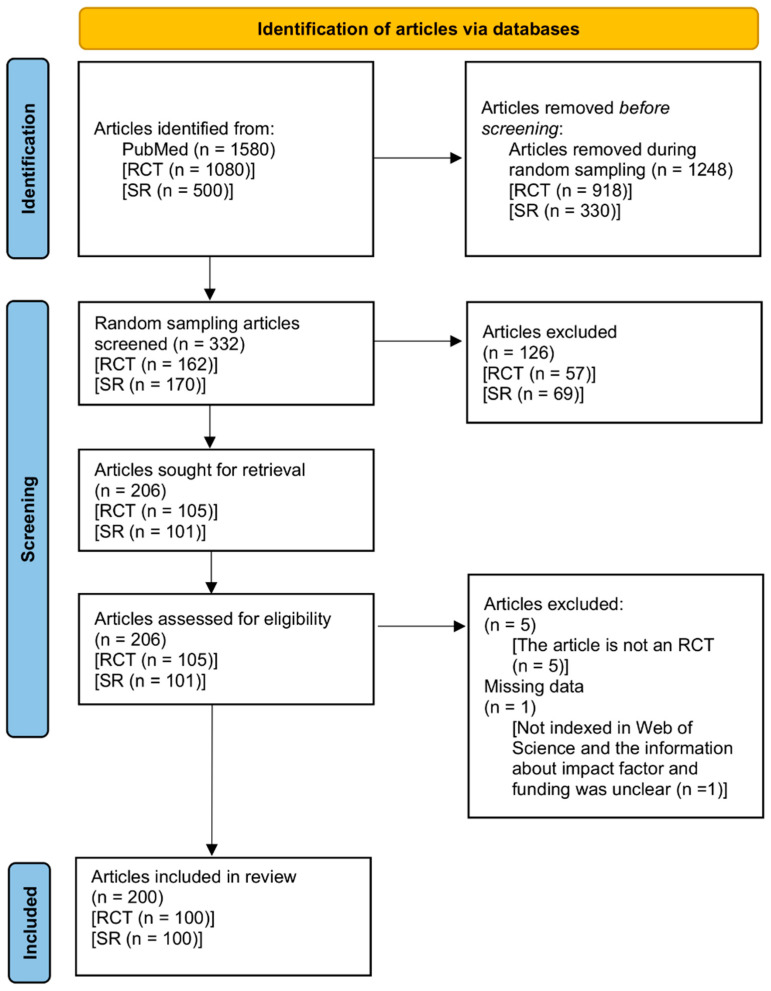
PRISMA 2020 flow diagram. Abbreviations: RCT, randomized controlled trial; SR, systematic review [28].

Figure 1 shows the PRISMA 2020 flow diagram used in this study. In total, 1580 full-text articles were retrieved from PubMed. We obtained 332 random sample articles (RCT, *n* = 162 and SR, *n* = 170). We excluded 126 articles (RCT, *n* = 57 and SR, *n* = 69) during title and abstract screening. Then, we screened 206 full texts (RCT, *n* = 105 and SR, *n* = 101) and excluded five articles for reasons (Supplementary Materials, Text S1). Among those, we excluded one article from the statistical analyses because it was not indexed on the Web of Science, and the information about impact factors and funding was unclear (Supplementary Materials, Text S1). Ultimately, 100 RCTs and 100 SRs were included in the study. We listed the excluded studies in the Supplementary Materials, Text S1 and the included studies in the Supplementary Materials, Texts S2 and S3.

### 3.2. Proportions and Trends of RCT and SR Abstracts in Which Insomnia Concept Was Unclear

The unclear insomnia concept accounted for 88% of the RCT and 94% of the SR abstracts. Table S3 visually shows no clear trend for years of publication (*p*-values for trend for RCT and SR were 0.007 and 0.19, respectively). The proportions of included RCT and SR articles with abstract word counts below the upper limit in the instructions for the authors were 35% and 74% in abstracts with the unclear insomnia concept, whereas the proportions were 17% and 67% in abstracts with the clear insomnia concept, respectively.

### 3.3. The Percentage of Unclear Insomnia Concept of RCT and SR Abstracts If We Consider What Was Stated in the Full Text

Among the RCT and SR abstracts with unclear definitions, Table 1 shows the percentage of unclear insomnia concept of the abstracts, based on the description of methods and results in the full text. We could not classify the insomnia concept in 27% of the RCTs and 57% of the SRs following full-text review.

Table S4 shows that among the RCT and SR abstracts with the unclear insomnia concept, when insomnia meant insomnia disorder (total of insomnia disorder and both insomnia disorder and insomnia symptoms), diagnostic criteria were not reported in 14% of RCTs and 41% of SRs. In addition, 67% of the RCTs and 94% of the SRs did not report what type of person in what occupation diagnosed an insomnia disorder.

### 3.4. Characteristics of RCT and SR Abstracts with Unclear Insomnia Concept, and the Relationship between the Characteristics and Unclear Definitions in Abstracts

Table 2 shows the characteristics of RCT and SR abstracts in which the concept of insomnia was unclear and clear. No variables showed statistically significant differences using Fisher’s exact or Mann–Whitney U tests.

The unclear insomnia concept in RCT and SR abstracts was not significantly associated with any characteristics in the multivariable logistic regression analysis (Table 3).

## 4. Discussion

Approximately 90% of the RCTs and SRs, in which the word insomnia was mentioned in the methods or results in the abstract, did not distinguish between insomnia disorder and insomnia symptoms. Even when reviewing the descriptions of methods or results in the full texts, we determined that the insomnia concept (including detailed information about the diagnosis for insomnia disorder, for example, diagnostic criteria and the profession of the diagnostician) was unclear in 27% of RCTs and in 57% of SRs. We could not find any characteristics related to the lack of clarity of the abstracts.

The unclear concept of insomnia in RCT and SR abstracts is a pervasive problem because 90% of abstracts cannot determine whether insomnia is a diagnosis or a symptom. This result was consistent with a previous study that found that the quality of reporting of methods and results in abstracts of RCTs at sleep medicine conferences was inadequate [3]. Even when looking at the full text, it was often difficult to determine the difference between diagnosis and symptoms. The authors of RCTs and SRs used the term insomnia with ambiguous understanding. In addition, word limitations of abstracts would not be a reason for the lack of clarity because our results showed room for word counts in many RCT and SR abstracts. The authors of RCTs and SRs are encouraged to clearly state “insomnia disorder” or “insomnia symptoms” in the methods and results of the abstract and full text. In addition, the reviewer/editor should note the lack of clarity in the peer review process. In the future, editors should be instructed to include “to clearly state ”insomnia disorder” or ”insomnia symptoms” in the methods and results of the abstract and full text” as a guideline/criterion in reviewing articles on this topic (especially in journals in the “sleep” field). In addition, the authors of RCTs and SRs should provide a detailed description of the diagnosis of insomnia disorders in the full text.

This study did not clarify the characteristics of RCT and SR abstracts related to the unclear concept of insomnia in abstracts. Funding was not associated with the unclear concept of insomnia in the RCTs and SRs. The result was inconsistent in that funding was associated with the reporting quality of RCT abstracts in a previous study [3]. A possible reason for this inconsistency was that the population in this study was different from that in the previous study (46% of the abstracts in the previous study were on topics other than insomnia) [3]. Another possible reason for the inconsistency is that the abstracts in this study had undergone a peer-review process, whereas the abstracts of conference proceedings in the previous study may not have undergone a peer-review process [3].

This study had several limitations. First, the sample size was inadequate for statistical analysis because many RCTs and SRs were unclear about the concept of insomnia in the abstracts. Due to the small sample size, we believe that caution should be exercised when considering the year of publication as a relevant factor for the unclear insomnia concept. Second, the results of this study may not apply to articles from PubMed indexing because we searched only PubMed. The proportion of unclear insomnia concept might be higher in RCT and SR abstracts from low-quality journals that were not indexed in PubMed. Third, we did not evaluate whether the authors of the RCTs and SRs included psychiatrists or clinical psychologists. To overcome this limitation, we included the journal name factor, which included sleep, in our analysis.

## 5. Conclusions

The concept of insomnia was unclearly stated in most RCT and SR abstracts. This study should be updated periodically in the future to observe whether the proportion of RCT and SR abstracts with a clear concept of insomnia increases. Authors of RCTs and SRs are recommended to state “insomnia disorder” or “insomnia symptoms” in the methods and results of abstracts. The reviewer/editor should point this out in the peer review if the authors do not state this.

## Figures and Tables

**Table 1:** The percentage of unclear insomnia concept of RCT and SR abstracts based on full-text articles.

Category	Subcategory	RCT n = 88	SR n = 94
Intended insomnia concept	Unclear insomnia concept	24 (27)	54 (57)
	Insomnia disorder	5 (6)	12 (13)
	Insomnia symptoms	43 (49)	23 (24)
	Both insomnia disorder and insomnia symptoms	16 (18)	5 (5)

Abbreviations: RCT, randomized controlled trial; SR, systematic review. Values are given as numbers (percentages). The denominator was the number of RCT and SR abstracts with the unclear insomnia concept.

**Table 2:** Characteristics of RCT and SR abstracts in which the insomnia concept is unclear and clear.

Category	Subcategory	RCTs		SRs	
		Unclear Insomnia Concept n = 88	Clear Insomnia Concept n = 12	Unclear Insomnia Concept n = 94	Clear Insomnia Concept n = 6
Impact Factor	0 to 4	58 (66)	10 (83)	46 (49)	4 (67)
	5 to 9	17 (19)	1 (8)	35 (37)	1 (17)
	10 or more	8 (9)	1 (8)	4 (4)	1 (17)
	No value	5 (6)	0 (0)	9 (10)	0 (0)
Word counts in abstracts		median 270 (IQR 244 to 311)	median 288 (IQR 272 to 298)	median 279 (IQR 237 to 387)	median 246 (IQR 241 to 257)
Word limitations in abstracts	Less than 300	64 (73)	9 (75)	39 (41)	4 (67)
	300 or more	20 (23)	2 (17)	43 (46)	1 (17)
	Unclear	4 (5)	1 (8)	12 (13)	1 (17)
Funding	None	12 (14)	1 (8)	53 (56)	3 (50)
	Industry	29 (33)	5 (42)	3 (3)	0 (0)
	Non-industry	47 (53)	6 (50)	38 (40)	3 (50)
Compliance with reporting guidelines	No compliance	77 (88)	12 (100)	46 (49)	3 (50)
	Compliance	11 (13)	0 (0)	48 (51)	3 (50)
Registrations or protocols	No registrations/protocols	21 (24)	4 (33)	63 (67)	6 (100)
	Registrations/protocols	67 (76)	8 (67)	31 (33)	0 (0)
Journal names that included sleep	Non-sleep journal	68 (77)	9 (75)	90 (96)	5 (83)
	Sleep journal	20 (23)	3 (25)	4 (4)	1 (17)

Abbreviations: RCT, randomized controlled trial; SR, systematic review; IQR, interquartile range; NA, not applicable. Values are presented as numbers (percentages). The definitions of the variables are presented in Table S2.

**Table 3:** Factors associated with RCT and SR abstracts in which the insomnia concept is unclear.

Category	Subcategory	RCTs (n = 100)		SRs (n = 100)	
		Crude OR (95% CI)	AOR (95% CI)	Crude OR (95% CI)	AOR (95% CI)
Impact factor	0 to 4	Ref	Ref	Ref	Ref
	5 to 9	2.09 (0.35, 12.57)	1.97 (0.33, 11.81)	2.29 (0.34, 15.29)	1.69 (0.23, 12.55)
	10 or more	1.02 (0.16, 6.51)	1.43 (0.14, 14.26)	0.29 (0.04, 2.34)	0.50 (0.05, 4.99)
	No value	1.97 (0.10, 38.43)	4.09 (0.11, 149.78)	1.84 (0.09, 37.08)	4.22 (0.15, 121.40)
Word counts in abstracts		1.00 (0.99, 1.01)	1.00 (0.99, 1.01)	1.00 (0.99, 1.01)	1.00 (1.00, 1.01)
Word limitations in abstracts	Less than 300	Ref	Ref	Ref	Ref
	300 or more	1.21 (0.28, 5.30)	1.44 (0.28, 7.42)	3.30 (0.50, 22.03)	2.18 (0.28, 16.92)
	Unclear	0.44 (0.06, 3.17)	0.31 (0.03, 3.44)	0.95 (0.13, 6.70)	0.63 (0.07, 5.44)
Funding	None	Ref	Ref	Ref	Ref
	Industry	0.64 (0.09, 4.40)	0.71 (0.10, 5.33)	0.46 (0.02, 10.73)	0.77 (0.03, 22.63)
	Non-industry	0.88 (0.13, 5.76)	0.79 (0.11, 5.90)	0.72 (0.15, 3.35)	0.79 (0.17, 3.64)
Compliance with reporting guidelines	No compliance	Ref	Ref	Ref	Ref
	Compliance	3.71 (0.21, 67.00)	3.05 (0.16, 57.19)	1.04 (0.22, 4.84)	0.87 (0.19, 4.02)
Registrations or protocols	No registrations/protocols	Ref	Ref	Ref	Ref
	Registrations/protocols	1.66 (0.48, 5.75)	1.60 (0.40, 6.35)	6.45 (0.35, 118.14)	6.40 (0.36, 114.28)
Journal names that included sleep	Non-sleep journal	Ref	Ref	Ref	Ref
	Sleep journal	0.81 (0.22, 3.04)	0.77 (0.18, 3.30)	0.18 (0.02, 1.40)	0.38 (0.03, 4.42)

Abbreviations: RCT, randomized controlled trial; SR, systematic review; OR, odds ratio; CI, confidence interval; AOR, adjusted odds ratio; Ref, reference. The definitions of variables were written in Table S2.

## Data Availability

The data presented in this study are available in Tables S5 and S6.

## Supplementary Materials

The following supporting information can be downloaded at: https://www.mdpi.com/article/10.3390/ijerph191912261/s1. Table S1: PRISMA 2020 Checklist, Table S2: Variable table for assessment of the proportion and characteristics that did not describe insomnia concept clearly in abstracts of RCTs and SRs, Table S3: The proportion of RCT and SR abstracts in which the insomnia concept is unclear, Table S4: Diagnosis of insomnia disorder used in the methods or results of the full text, Table S5: The raw data for randomized controlled trials, Table S6: The raw data for systematic reviews, Text S1: The reference list for the excluded articles, Text S2: The reference list for the included randomized controlled trials, Text S3: The reference list for the included systematic reviews.
