# Supplementary material for: Unclear Insomnia Concept in Randomized Controlled Trials and Systematic Reviews: A Meta-Epidemiological Study
Source: Int J Environ Res Public Health. 2022 Sep 27;19(19):12261. doi: 10.3390/ijerph191912261 (PMC9566752; doi:10.3390/ijerph191912261)
Supplement: Supplementary file 1 [file ijerph-19-12261-s001.zip › Supplementary materials.pdf]

Table S1. PRISMA 2020 Checklist

Section and Topic	Item #	Checklist item	Location where item is reported
**TITLE**			
Title	1	Identify the report as a systematic review.	Not applicable
**ABSTRACT**			
Abstract	2	See the PRISMA 2020 for Abstracts checklist.	1
**INTRODUCTION**			
Rationale	3	Describe the rationale for the review in the context of existing knowledge.	2
Objectives	4	Provide an explicit statement of the objective(s) or question(s) the review addresses.	2
**METHODS**			
Eligibility criteria	5	Specify the inclusion and exclusion criteria for the review and how studies were grouped for the syntheses.	3
Information sources	6	Specify all databases, registers, websites, organisations, reference lists and other sources searched or consulted to identify studies. Specify the date when each source was last searched or consulted.	3
Search strategy	7	Present the full search strategies for all databases, registers and websites, including any filters and limits used.	3
Selection process	8	Specify the methods used to decide whether a study met the inclusion criteria of the review, including how many reviewers screened each record and each report retrieved, whether they worked independently, and if applicable, details of automation tools used in the process.	3
Data collection process	9	Specify the methods used to collect data from reports, including how many reviewers collected data from each report, whether they worked independently, any processes for obtaining or confirming data from study investigators, and if applicable, details of automation tools used in the process.	3
Data items	10a	List and define all outcomes for which data were sought. Specify whether all results that were compatible with each outcome domain in each study were sought (e.g. for all measures, time points, analyses), and if not, the methods used to decide which results to collect.	Table S2

Section and Topic	Item #	Checklist item	Location where item is reported
	10b	List and define all other variables for which data were sought (e.g. participant and intervention characteristics, funding sources). Describe any assumptions made about any missing or unclear information.	Table S2
Study risk of bias assessment	11	Specify the methods used to assess risk of bias in the included studies, including details of the tool(s) used, how many reviewers assessed each study and whether they worked independently, and if applicable, details of automation tools used in the process.	Not applicable
Effect measures	12	Specify for each outcome the effect measure(s) (e.g. risk ratio, mean difference) used in the synthesis or presentation of results.	4
Synthesis methods	13a	Describe the processes used to decide which studies were eligible for each synthesis (e.g. tabulating the study intervention characteristics and comparing against the planned groups for each synthesis (item #5)).	4
	13b	Describe any methods required to prepare the data for presentation or synthesis, such as handling of missing summary statistics, or data conversions.	Not applicable
	13c	Describe any methods used to tabulate or visually display results of individual studies and syntheses.	Not applicable
	13d	Describe any methods used to synthesize results and provide a rationale for the choice(s). If meta-analysis was performed, describe the model(s), method(s) to identify the presence and extent of statistical heterogeneity, and software package(s) used.	Not applicable
	13e	Describe any methods used to explore possible causes of heterogeneity among study results (e.g. subgroup analysis, meta-regression).	Not applicable
	13f	Describe any sensitivity analyses conducted to assess robustness of the synthesized results.	Not applicable
Reporting bias assessment	14	Describe any methods used to assess risk of bias due to missing results in a synthesis (arising from reporting biases).	Not applicable
Certainty assessment	15	Describe any methods used to assess certainty (or confidence) in the body of evidence for an outcome.	Not applicable
**RESULTS**			

Section and Topic	Item #	Checklist item	Location where item is reported
Study selection	16a	Describe the results of the search and selection process, from the number of records identified in the search to the number of studies included in the review, ideally using a flow diagram.	5, 6
	16b	Cite studies that might appear to meet the inclusion criteria, but which were excluded, and explain why they were excluded.	Text S1
Study characteristics	17	Cite each included study and present its characteristics.	Text S2 and S3, Table 2
Risk of bias in studies	18	Present assessments of risk of bias for each included study.	Not applicable
Results of individual studies	19	For all outcomes, present, for each study: (a) summary statistics for each group (where appropriate) and (b) an effect estimate and its precision (e.g. confidence/credible interval), ideally using structured tables or plots.	6 to 9
Results of syntheses	20a	For each synthesis, briefly summarise the characteristics and risk of bias among contributing studies.	Not applicable
	20b	Present results of all statistical syntheses conducted. If meta-analysis was done, present for each the summary estimate and its precision (e.g. confidence/credible interval) and measures of statistical heterogeneity. If comparing groups, describe the direction of the effect.	Not applicable
	20c	Present results of all investigations of possible causes of heterogeneity among study results.	Not applicable
	20d	Present results of all sensitivity analyses conducted to assess the robustness of the synthesized results.	Not applicable
Reporting biases	21	Present assessments of risk of bias due to missing results (arising from reporting biases) for each synthesis assessed.	Not applicable
Certainty of evidence	22	Present assessments of certainty (or confidence) in the body of evidence for each outcome assessed.	Not applicable
**DISCUSSION**			

Section and Topic	Item #	Checklist item	Location where item is reported
Discussion	23a	Provide a general interpretation of the results in the context of other evidence.	10
	23b	Discuss any limitations of the evidence included in the review.	10
	23c	Discuss any limitations of the review processes used.	10
	23d	Discuss implications of the results for practice, policy, and future research.	10, 11
**OTHER INFORMATION**			
Registration and protocol	24a	Provide registration information for the review, including register name and registration number, or state that the review was not registered.	Not applicable
	24b	Indicate where the review protocol can be accessed, or state that a protocol was not prepared.	3
	24c	Describe and explain any amendments to information provided at registration or in the protocol.	4, 5
Support	25	Describe sources of financial or non-financial support for the review, and the role of the funders or sponsors in the review.	11
Competing interests	26	Declare any competing interests of review authors.	11
Availability of data, code and other materials	27	Report which of the following are publicly available and where they can be found: template data collection forms; data extracted from included studies; data used for all analyses; analytic code; any other materials used in the review.	11

From: Page MJ, McKenzie JE, Bossuyt PM, Boutron I, Hoffmann TC, Mulrow CD, et al. The PRISMA 2020 statement: an updated guideline for reporting systematic reviews. BMJ 2021;372:n71. doi: 10.1136/bmj.n71

Abbreviation: PRISMA, Preferred Reporting Items for Systematic Reviews and Meta-Analyses

Table S2. Variable table for assessment of the proportion and characteristics that did not describe insomnia concept clearly in abstracts of RCTs and SRs

Variables (Category)	Definition
Insomnia concepts in abstracts (clear insomnia concept, unclear insomnia concept)	Clear insomnia concept: the number of RCT and SR articles that can be distinguished betw een insomnia disorder and insomnia symptoms from the description in abstracts.
Year of publication	The years in w hich eligible RCTs and SRs w ere published. We excerpted years of print publication. We excerpted years of electronic publication if articles have not yet been published in print publication.
Impact factor (0 to 4, 5 to 9, 10 or more, and no value)	Impact factors in the journal in w hich the review w as published. We excerpted Journal Impact Factor 2020 of the journal in the Journal Citation Reports 2021. We treated the follow ing numerical variables as categorical variables: impact factor (0 to 4, 5 to 9, 10 or more, and no value).
Word count in abstracts	The number of w ords in abstracts
Word limitation in abstracts (less than 300, 300 or more, unclear)	The number of maximum w ords about abstracts. We excerpted the information in instructions for the authors of each journal. We recorded "unclear" if the instructions for the authors of each journal did not mention the number of maximum w ords about abstracts.
Funding (industry, non-industry, none, or unclear)	We excerpted information about funding from full texts in eligible articles and from articles on the Web of Science. First, the funding is coded based on the funding information extracted from the full text. Next, if the funding is determined to be "unclear" or "none" based on the information in the full text, and if the funding is classified as "non-industry" or "industry" based on the funding information extracted from the Web of Science, the funding information from the Web of Science is used.<br>Industry: either industry funding only (the funds w ere received from industries) or both industry and non-industry funding<br>Non-industry: the funds w ere from governments and other academic or nonprofit organizations.<br>None: the eligible articles had no funding.<br>Unclear: no information about funding
Compliance w ith reporting guidelines (no compliance, compliance)	No compliance: methods in full texts did not mention that eligible articles w ere referring to reporting guidelines.<br>Compliance: methods in full texts mentioned eligible articles w ere referring to reporting guidelines (CONSORT for RCTs, and PRISMA or MOOSE for SRs)
Registrations or protocols (no registrations/ protocols, registrations/ protocols)	No registrations/ protocols: other than “registrations/ protocols”<br>Registrations/ protocols: methods in full texts mentioned information about clinical trials registrations or SR registrations or protocols for RCTs or SRs
Journal name w hich included sleep (non-sleep journal, sleep journal)	Non-sleep journal: Other than “sleep journal”. Sleep journal: The name of the journal included the w ord “sleep”

Abbreviation: CONSORT, Consolidated Standards of Reporting Trials; MOOSE, Meta-analysis Of Observational Studies in Epidemiology; PRISMA, Preferred Reporting Items for Systematic Reviews and Meta-Analyses; RCTs, randomized controlled trials; SRs, systematic reviews

The denominator was the number of RCT and SR articles that mentioned insomnia in methods or results of abstracts.

Table S3. The proportion of RCTs and SRs abstracts in which the insomnia concept is unclear

Year	2010	2011	2012	2013	2014	2015	2016	2017	2018	2019	2020	2021	2022
Unclear insomnia concept in RCT (n = 88)	5 (56)	5 (100)	2 (67)	7 (100)	6 (67)	5 (100)	6 (86)	5 (83)	16 (100)	10 (91)	5 (83)	14 (100)	2 (100)
Unclear insomnia concept in SR (n = 94)	NA[a]	1 (100)	4 (100)	2 (100)	6 (100)	5 (100)	8 (100)	8 (89)	5 (100)	12 (92)	19 (95)	16 (84)	8 (100)

Abbreviations: RCT, randomized controlled trial; SR, systematic review; NA, not applicable

Note: Values are given as a number (percentage). The denominator of proportion is the number of RCTs or SRs abstracts published each year.

a The 100 random samples for SRs did not include SRs published in 2010.

Table S4. Diagnosis of insomnia disorder used in the methods or results of the full text

Category	Subcategory	RCT n = 21	SR n = 17
Diagnostic criteria	Not reported	3 (14)	7 (41)
	DSM	12 (57)	4 (24)
	ICD	1 (5)	0 (0)
	ICSD	3 (14)	1 (6)
	other	2 (10)	5 (29)
Who diagnosed the insomnia disorder	Not reported	14 (67)	16 (94)
	Clinician	7 (33)	0 (0)
	Researcher	0 (0)	0 (0)
	Both clinician and researcher	0 (0)	1 (6)

Abbreviations: RCT, randomized controlled trial; SR, systematic review; DSM, The Diagnostic and Statistical Manual of Mental Disorders; ICD, International Classification of Diseases; ICSD, The International Classification of Sleep Disorders

Values are given as a number (percentage).

The denominator was the number of RCTs and SRs abstracts with the unclear insomnia concept and with the the intention of insomnia disorder based on the description of full text.

We coded “other” in diagnostic criteria if two or more standard criteria, DSM, ICD, ICSD, were used, or diagnostic criteria other than the standard criteria was used.

Text S1. The reference list for the excluded articles

1. Balk E, A.G., Kimmel H, Rofeberg V, Saeed I, Jeppson P, Trikalinos T. *Nonsurgical Treatments for Urinary Incontinence in Women: A Systematic Review Update [Internet]*; Agency for Healthcare Research and Quality (US): Rockville (MD), 2018.

2. Campana, L.M.; Clifford, G.D.; Trinder, J.; Pittman, S.D.; Malhotra, A. A possible method to predict response to non-pharmacological insomnia therapy. *J Clin Sleep Med* **2011**, *7*, 370-375, doi:10.5664/JCSM.1192.
3. Mittal, D.; Fortney, J.C.; Pyne, J.M.; Wetherell, J.L. Predictors of persistence of comorbid generalized anxiety disorder among veterans with major depressive disorder. *J Clin Psychiatry* **2011**, *72*, 1445-1451, doi:10.4088/JCP.10m05981blu.
4. Ogawa, A.; Hinotsu, S.; Urushihara, H.; Kawakami, K. Night-to-night variability of sleep latency significantly predicts the magnitude of subsequent change in sleep latency during placebo administration. *Sleep Med* **2011**, *12*, 565-571, doi:10.1016/j.sleep.2011.03.009.
5. Rumble, M.E.; McCall, W.V.; Dickson, D.A.; Krystal, A.D.; Rosenquist, P.B.; Benca, R.M. An exploratory analysis of the association of circadian rhythm dysregulation and insomnia with suicidal ideation over the course of treatment in individuals with depression, insomnia, and suicidal ideation. *J Clin Sleep Med* **2020**, *16*, 1311-1319, doi:10.5664/jcsm.8508.
6. Tsai, H.W.; Wang, P.H.; Huang, B.S.; Twu, N.F.; Yen, M.S.; Chen, Y.J. Low-dose add-back therapy during postoperative GnRH agonist treatment. *Taiwan J Obstet Gynecol* **2016**, *55*, 55-59, doi:10.1016/j.tjog.2015.04.004.

The reason for exclusion for reference 1 was not indexed in the Web of Science.

The reasons for exclusion for references 2, 3, 4, 5, and 6 were these reports were not randomized controlled trials.

Text S2. The reference list for the included randomized controlled trials

1. Akodu, A.K.; Nwanne, C.A.; Fapojuwo, O.A. Efficacy of neck stabilization and Pilates exercises on pain, sleep disturbance and kinesiophobia in patients with non-specific chronic neck pain: A randomized controlled trial. *J Bodyw Mov Ther* **2021**, *26*, 411-419, doi:10.1016/j.jbmt.2020.09.008.
2. Alshehri, M.M.; Alenazi, A.M.; Alothman, S.A.; Rucker, J.L.; Phadnis, M.A.; Miles, J.M.; Siengsukon, C.F.; Kluding, P.M. Using Cognitive Behavioral Therapy for Insomnia in People with Type 2 Diabetes, Pilot RCT Part I: Sleep and Concomitant Symptom. *Behav Sleep Med* **2021**, *19*, 652-671, doi:10.1080/15402002.2020.1831501.
3. Alshehri, M.M.; Alothman, S.A.; Alenazi, A.M.; Rucker, J.L.; Phadnis, M.A.; Miles, J.M.; Siengsukon, C.F.; Kluding, P.M. The effects of cognitive behavioral therapy for insomnia in people with type 2 diabetes mellitus, pilot RCT part II: diabetes health outcomes. *BMC Endocr Disord* **2020**, *20*, 136, doi:10.1186/s12902-020-00612-6.
4. Arnold, L.M.; Hirsch, I.; Sanders, P.; Ellis, A.; Hughes, B. Safety and efficacy of esreboxetine in patients with fibromyalgia: a fourteen-week, randomized, double-blind, placebo-controlled, multicenter clinical trial. *Arthritis Rheum* **2012**, *64*, 2387-2397,

doi:10.1002/art.34390.
5. Aziz, S.; Qamar, R.; Ahmed, I.; Imran, K.; Masroor, M.; Rajper, J.; Nafay, S.; Noorulain, W.; Khan, M.H. Treatment profile of hepatitis C patients - a comparison of interferon alpha 2a and 2b treatment regimes. *J Coll Physicians Surg Pak* **2010**, *20*, 581-585, doi:09.2010/JCPSP.581585.
6. Bao, T.; Baser, R.; Chen, C.; Weitzman, M.; Zhang, Y.L.; Seluzicki, C.; Li, Q.S.; Piulson, L.; Zhi, W.I. Health-Related Quality of Life in Cancer Survivors with Chemotherapy-Induced Peripheral Neuropathy: A Randomized Clinical Trial. *Oncologist* **2021**, *26*, e2070-e2078, doi:10.1002/onco.13933.
7. Beaulieu-Bonneau, S.; Ivers, H.; Guay, B.; Morin, C.M. Long-Term Maintenance of Therapeutic Gains Associated With Cognitive-Behavioral Therapy for Insomnia Delivered Alone or Combined With Zolpidem. *Sleep* **2017**, *40*, doi:10.1093/sleep/zsx002.
8. Behrendt, D.; Ebert, D.D.; Spiegelhalder, K.; Lehr, D. Efficacy of a Self-Help Web-Based Recovery Training in Improving Sleep in Workers: Randomized Controlled Trial in the General Working Population. *J Med Internet Res* **2020**, *22*, e13346, doi:10.2196/13346.
9. Bentley, T.G.K.; Castillo, D.; Sadeghi, N.; Piber, D.; Carroll, J.; Olmstead, R.; Irwin, M.R. Costs associated with treatment of insomnia in Alzheimer's disease caregivers: a comparison of mindfulness meditation and cognitive behavioral therapy for insomnia. *BMC Health Serv Res* **2022**, *22*, 231, doi:10.1186/s12913-022-07619-w.
10. Bicego, A.; Monseur, J.; Collinet, A.; Donneau, A.F.; Fontaine, R.; Libbrecht, D.; Malaise, N.; Nyssen, A.S.; Raaf, M.; Rousseaux, F.; et al. Complementary treatment comparison for chronic pain management: A randomized longitudinal study. *PLoS One* **2021**, *16*, e0256001, doi:10.1371/journal.pone.0256001.
11. Bondi, C.D.; Kamal, K.M.; Johnson, D.A.; Witt-Enderby, P.A.; Giannetti, V.J. The Effect of Melatonin Upon Postacute Withdrawal Among Males in a Residential Treatment Program (M-PAWS): A Randomized, Double-blind, Placebo-controlled Trial. *J Addict Med* **2018**, *12*, 201-206, doi:10.1097/ADM.0000000000000386.
12. Borghi, B.; Aurini, L.; White, P.F.; Tognu, A.; Rossi, B.; Fini, G.; Rucci, P.; Greggi, T.; Borghi, R. Treatment of recent onset low back pain with periradicular injections of meloxicam: a randomized, double blind, placebo controlled cross-over study. *Minerva Anestesiol* **2018**, *84*, 590-598, doi:10.23736/S0375-9393.18.12221-8.
13. Buysse, D.J.; Germain, A.; Moul, D.E.; Franzen, P.L.; Brar, L.K.; Fletcher, M.E.; Begley, A.; Houck, P.R.; Mazumdar, S.; Reynolds, C.F., 3rd; et al. Efficacy of brief behavioral treatment for chronic insomnia in older adults. *Arch Intern Med* **2011**, *171*, 887-895, doi:10.1001/archinternmed.2010.535.
14. Childress, A.C.; Arnold, V.; Adeyi, B.; Dirks, B.; Babcock, T.; Scheckner, B.; Lasser, R.; Lopez, F.A. The effects of lisdexamfetamine dimesylate on emotional lability in children 6 to 12 years of age with ADHD in a double-blind placebo-controlled trial. *J Atten Disord* **2014**, *18*, 123-132, doi:10.1177/1087054712448252.
15. Childress, A.C.; Kollins, S.H.; Cutler, A.J.; Marraffino, A.; Sikes, C.R. Efficacy, Safety, and Tolerability of an Extended-Release Orally Disintegrating Methylphenidate Tablet in Children 6-12 Years of Age with Attention-Deficit/Hyperactivity Disorder in the Laboratory

Classroom Setting. *J Child Adolesc Psychopharmacol* **2017**, *27*, 66-74, doi:10.1089/cap.2016.0002.

16. Chollet, F.; Tardy, J.; Albucher, J.F.; Thalamas, C.; Berard, E.; Lamy, C.; Bejot, Y.; Deltour, S.; Jaillard, A.; Niclot, P.; et al. Fluoxetine for motor recovery after acute ischaemic stroke (FLAME): a randomised placebo-controlled trial. *Lancet Neurol* **2011**, *10*, 123-130, doi:10.1016/S1474-4422(10)70314-8.
17. Chung, K.F.; Yeung, W.F.; Yu, Y.M.; Yung, K.P.; Zhang, S.P.; Zhang, Z.J.; Wong, M.T.; Lee, W.K.; Chan, L.W. Acupuncture for residual insomnia associated with major depressive disorder: a placebo- and sham-controlled, subject- and assessor-blind, randomized trial. *J Clin Psychiatry* **2015**, *76*, e752-760, doi:10.4088/JCP.14m09124.
18. Clayton, A.H.; Croft, H.A.; Yuan, J.; Brown, L.; Kissling, R. Safety of Flibanserin in Women Treated With Antidepressants: A Randomized, Placebo-Controlled Study. *J Sex Med* **2018**, *15*, 43-51, doi:10.1016/j.jsxm.2017.11.005.
19. Cohen, L.S.; Joffe, H.; Guthrie, K.A.; Ensrud, K.E.; Freeman, M.; Carpenter, J.S.; Learman, L.A.; Newton, K.M.; Reed, S.D.; Manson, J.E.; et al. Efficacy of omega-3 for vasomotor symptoms treatment: a randomized controlled trial. *Menopause* **2014**, *21*, 347-354, doi:10.1097/GME.0b013e31829e40b8.
20. Colvonen, P.J.; Straus, L.D.; Drummond, S.P.A.; Angkaw, A.C.; Norman, S.B. Examining sleep over time in a randomized control trial comparing two integrated PTSD and alcohol use disorder treatments. *Drug Alcohol Depend* **2020**, *209*, 107905, doi:10.1016/j.drugalcdep.2020.107905.
21. Cornu, C.; Remontet, L.; Noel-Baron, F.; Nicolas, A.; Feugier-Favier, N.; Roy, P.; Claustrat, B.; Saadatian-Elahi, M.; Kassai, B. A dietary supplement to improve the quality of sleep: a randomized placebo controlled trial. *BMC Complement Altern Med* **2010**, *10*, 29, doi:10.1186/1472-6882-10-29.
22. Cutler, A.J.; Kalali, A.H.; Mattingly, G.W.; Kunovac, J.; Meng, X. Long-term safety and tolerability of iloperidone: results from a 25-week, open-label extension trial. *CNS Spectr* **2013**, *18*, 43-54, doi:10.1017/S1092852912000764.
23. Dos Reis Lucena, L.; Dos Santos-Junior, J.G.; Tufik, S.; Hachul, H. Lavender essential oil on postmenopausal women with insomnia: Double-blind randomized trial. *Complement Ther Med* **2021**, *59*, 102726, doi:10.1016/j.ctim.2021.102726.
24. Dreno, B.; Ascierto, P.A.; Atkinson, V.; Liszkay, G.; Maio, M.; Mandala, M.; Demidov, L.; Stroyakovskiy, D.; Thomas, L.; de la Cruz-Merino, L.; et al. Health-related quality of life impact of cobimetinib in combination with vemurafenib in patients with advanced or metastatic BRAF(V600) mutation-positive melanoma. *Br J Cancer* **2018**, *118*, 777-784, doi:10.1038/bjc.2017.488.
25. Duncan, M.J.; Fenton, S.; Brown, W.J.; Collins, C.E.; Glozier, N.; Kolt, G.S.; Holliday, E.G.; Morgan, P.J.; Murawski, B.; Plotnikoff, R.C.; et al. Efficacy of a Multi-component m-Health Weight-loss Intervention in Overweight and Obese Adults: A Randomised Controlled Trial. *Int J Environ Res Public Health* **2020**, *17*, doi:10.3390/ijerph17176200.
26. Durgam, S.; Starace, A.; Li, D.; Migliore, R.; Ruth, A.; Nemeth, G.; Laszlovszky, I. An evaluation of the safety and efficacy of cariprazine in patients with acute exacerbation of

schizophrenia: a phase II, randomized clinical trial. *Schizophr Res* **2014**, *152*, 450-457, doi:10.1016/j.schres.2013.11.041.
27. Ensrud, K.E.; Joffe, H.; Guthrie, K.A.; Larson, J.C.; Reed, S.D.; Newton, K.M.; Sternfeld, B.; Lacroix, A.Z.; Landis, C.A.; Woods, N.F.; et al. Effect of escitalopram on insomnia symptoms and subjective sleep quality in healthy perimenopausal and postmenopausal women with hot flashes: a randomized controlled trial. *Menopause* **2012**, *19*, 848-855, doi:10.1097/gme.0b013e3182476099.
28. Esteban, R.; Pineda, J.A.; Calleja, J.L.; Casado, M.; Rodriguez, M.; Turnes, J.; Morano Amado, L.E.; Morillas, R.M.; Forns, X.; Pascasio Acevedo, J.M.; et al. Efficacy of Sofosbuvir and Velpatasvir, With and Without Ribavirin, in Patients With Hepatitis C Virus Genotype 3 Infection and Cirrhosis. *Gastroenterology* **2018**, *155*, 1120-1127 e1124, doi:10.1053/j.gastro.2018.06.042.
29. Forsell, E.; Jernelov, S.; Blom, K.; Kraepelien, M.; Svanborg, C.; Andersson, G.; Lindefors, N.; Kaldo, V. Proof of Concept for an Adaptive Treatment Strategy to Prevent Failures in Internet-Delivered CBT: A Single-Blind Randomized Clinical Trial With Insomnia Patients. *Am J Psychiatry* **2019**, *176*, 315-323, doi:10.1176/appi.ajp.2018.18060699.
30. Frick, G.; Yan, B.; Adler, L.A. Triple-Bead Mixed Amphetamine Salts (SHP465) in Adults With ADHD: Results of a Phase 3, Double-Blind, Randomized, Forced-Dose Trial. *J Atten Disord* **2020**, *24*, 402-413, doi:10.1177/1087054717696771.
31. Fu, D.J.; Turkoz, I.; Simonson, R.B.; Walling, D.P.; Schooler, N.R.; Lindenmayer, J.P.; Canuso, C.M.; Alphs, L. Paliperidone palmitate once-monthly reduces risk of relapse of psychotic, depressive, and manic symptoms and maintains functioning in a double-blind, randomized study of schizoaffective disorder. *J Clin Psychiatry* **2015**, *76*, 253-262, doi:10.4088/JCP.14m09416.
32. Gaspar, M.P.; Osterman, M.N.; Shin, E.K.; Osterman, A.L.; Kane, P.M. Sleep disturbance and response to surgical decompression in patients with carpal tunnel syndrome: a prospective randomized pilot comparison of open versus endoscopic release. *Acta Biomed* **2019**, *90*, 92-96, doi:10.23750/abm.v90i1.6474.
33. Gehrman, P.; Gunter, P.; Findley, J.; Frasso, R.; Weljie, A.M.; Kuna, S.T.; Kayser, M.S. Randomized Noninferiority Trial of Telehealth Delivery of Cognitive Behavioral Treatment of Insomnia Compared to In-Person Care. *J Clin Psychiatry* **2021**, *82*, doi:10.4088/JCP.20m13723.
34. Glozier, N.; Christensen, H.; Griffiths, K.M.; Hickie, I.B.; Naismith, S.L.; Biddle, D.; Overland, S.; Thorndike, F.; Ritterband, L. Adjunctive Internet-delivered cognitive behavioural therapy for insomnia in men with depression: A randomised controlled trial. *Aust N Z J Psychiatry* **2019**, *53*, 350-360, doi:10.1177/0004867418797432.
35. Goerigk, S.A.; Padberg, F.; Chekroud, A.; Kambeitz, J.; Buhner, M.; Brunoni, A.R. Parsing the antidepressant effects of non-invasive brain stimulation and pharmacotherapy: A symptom clustering approach on ELECT-TDCS. *Brain Stimul* **2021**, *14*, 906-912, doi:10.1016/j.brs.2021.05.008.
36. Goral Turkcu, S.; Ozkan, S. The effects of reflexology on anxiety, depression and quality of life in patients with gynecological cancers with reference to Watson's theory of human

caring. *Complement Ther Clin Pract* **2021**, *44*, 101428, doi:10.1016/j.ctcp.2021.101428.
37. Harb, G.C.; Cook, J.M.; Phelps, A.J.; Gehrman, P.R.; Forbes, D.; Localio, R.; Harpaz-Rotem, I.; Gur, R.C.; Ross, R.J. Randomized Controlled Trial of Imagery Rehearsal for Posttraumatic Nightmares in Combat Veterans. *J Clin Sleep Med* **2019**, *15*, 757-767, doi:10.5664/jcsm.7770.
38. Holmqvist, M.; Vincent, N.; Walsh, K. Web- vs. telehealth-based delivery of cognitive behavioral therapy for insomnia: a randomized controlled trial. *Sleep Med* **2014**, *15*, 187-195, doi:10.1016/j.sleep.2013.10.013.
39. Inoue, Y.; Shimizu, T.; Hirata, K.; Uchimura, N.; Ishigooka, J.; Oka, Y.; Ikeda, J.; Tomida, T.; Hattori, N.; Rotigotine Trial, G. Efficacy and safety of rotigotine in Japanese patients with restless legs syndrome: a phase 3, multicenter, randomized, placebo-controlled, double-blind, parallel-group study. *Sleep Med* **2013**, *14*, 1085-1091, doi:10.1016/j.sleep.2013.07.007.
40. Irving, G.; Tanenberg, R.J.; Raskin, J.; Risser, R.C.; Malcolm, S. Comparative safety and tolerability of duloxetine vs. pregabalin vs. duloxetine plus gabapentin in patients with diabetic peripheral neuropathic pain. *Int J Clin Pract* **2014**, *68*, 1130-1140, doi:10.1111/ijcp.12452.
41. Ivgy-May, N.; Ruwe, F.; Krystal, A.; Roth, T. Esmirtazapine in non-elderly adult patients with primary insomnia: efficacy and safety from a randomized, 6-week sleep laboratory trial. *Sleep Med* **2015**, *16*, 838-844, doi:10.1016/j.sleep.2015.04.001.
42. Johnson, J.R.; Lossignol, D.; Burnell-Nugent, M.; Fallon, M.T. An open-label extension study to investigate the long-term safety and tolerability of THC/CBD oromucosal spray and oromucosal THC spray in patients with terminal cancer-related pain refractory to strong opioid analgesics. *J Pain Symptom Manage* **2013**, *46*, 207-218, doi:10.1016/j.jpainsymman.2012.07.014.
43. Kalmbach, D.A.; Cheng, P.; Arnedt, J.T.; Anderson, J.R.; Roth, T.; Fellman-Couture, C.; Williams, R.A.; Drake, C.L. Treating insomnia improves depression, maladaptive thinking, and hyperarousal in postmenopausal women: comparing cognitive-behavioral therapy for insomnia (CBTI), sleep restriction therapy, and sleep hygiene education. *Sleep Med* **2019**, *55*, 124-134, doi:10.1016/j.sleep.2018.11.019.
44. Kinoshita, T.; Tanigawa, T.; Maruyama, K.; Morimoto, K. The effects of bright light treatment via ear canals on quality of sleep and depressive mood among overworked employees: A randomized-controlled clinical trial. *Work* **2020**, *67*, 323-329, doi:10.3233/WOR-203282.
45. Lancee, J.; van Straten, A.; Morina, N.; Kaldo, V.; Kamphuis, J.H. Guided Online or Face-to-Face Cognitive Behavioral Treatment for Insomnia: A Randomized Wait-List Controlled Trial. *Sleep* **2016**, *39*, 183-191, doi:10.5665/sleep.5344.
46. Landbloom, R.; Mackle, M.; Wu, X.; Kelly, L.; Snow-Adami, L.; McIntyre, R.S.; Mathews, M.; Hundt, C. Asenapine for the treatment of adults with an acute exacerbation of schizophrenia: results from a randomized, double-blind, fixed-dose, placebo-controlled trial with olanzapine as an active control. *CNS Spectr* **2017**, *22*, 333-341, doi:10.1017/S1092852916000377.

47. Leeangkoonsathian, E.; Pantasri, T.; Chaovisitseree, S.; Morakot, N. The effect of different progestogens on sleep in postmenopausal women: a randomized trial. *Gynecol Endocrinol* **2017**, *33*, 933-936, doi:10.1080/09513590.2017.1333094.
48. Maarrawi, J.; Abdel Hay, J.; Kobaiter-Maarrawi, S.; Tabet, P.; Peyron, R.; Garcia-Larrea, L. Randomized double-blind controlled study of bedtime low-dose amitriptyline in chronic neck pain. *Eur J Pain* **2018**, *22*, 1180-1187, doi:10.1002/ejp.1206.
49. Margolies, S.O.; Rybarczyk, B.; Vrana, S.R.; Leszczyszyn, D.J.; Lynch, J. Efficacy of a cognitive-behavioral treatment for insomnia and nightmares in Afghanistan and Iraq veterans with PTSD. *J Clin Psychol* **2013**, *69*, 1026-1042, doi:10.1002/jclp.21970.
50. McCall, W.V.; Benca, R.M.; Rosenquist, P.B.; Riley, M.A.; Hodges, C.; Gubosh, B.; McCloud, L.; Newman, J.C.; Case, D.; Rumble, M.; et al. A multi-site randomized clinical trial to reduce suicidal ideation in suicidal adult outpatients with Major Depressive Disorder: Development of a methodology to enhance safety. *Clin Trials* **2015**, *12*, 189-198, doi:10.1177/1740774515573958.
51. McCall, W.V.; Pillai, A.; Case, D.; McCloud, L.; Nolla, T.; Branch, F.; Youssef, N.A.; Moraczewski, J.; Tauhidul, L.; Pandya, C.D.; et al. A Pilot, Randomized Clinical Trial of Bedtime Doses of Prazosin Versus Placebo in Suicidal Posttraumatic Stress Disorder Patients With Nightmares. *J Clin Psychopharmacol* **2018**, *38*, 618-621, doi:10.1097/JCP.0000000000000968.
52. McCurry, S.M.; Shortreed, S.M.; Von Korff, M.; Balderson, B.H.; Baker, L.D.; Rybarczyk, B.D.; Vitiello, M.V. Who benefits from CBT for insomnia in primary care? Important patient selection and trial design lessons from longitudinal results of the Lifestyles trial. *Sleep* **2014**, *37*, 299-308, doi:10.5665/sleep.3402.
53. Mitchell, P.B.; Hadzi-Pavlovic, D.; Evoniuk, G.; Calabrese, J.R.; Bowden, C.L. A factor analytic study in bipolar depression, and response to lamotrigine. *CNS Spectr* **2013**, *18*, 214-224, doi:10.1017/S1092852913000291.
54. Newton, K.M.; Reed, S.D.; Guthrie, K.A.; Sherman, K.J.; Booth-LaForce, C.; Caan, B.; Sternfeld, B.; Carpenter, J.S.; Learman, L.A.; Freeman, E.W.; et al. Efficacy of yoga for vasomotor symptoms: a randomized controlled trial. *Menopause* **2014**, *21*, 339-346, doi:10.1097/GME.0b013e31829e4baa.
55. Ni, J.; Wang, F.; Wang, B.; Zhou, H.; Zhang, N.; Shi, H.; Xie, Q.; Zhao, H. Effectiveness and safety of auricular acupoint bloodletting in treatment of insomnia: an assessor-blinded pilot randomized controlled trial. *J Tradit Chin Med* **2018**, *38*, 763-768.
56. Peoples, A.R.; Garland, S.N.; Pigeon, W.R.; Perlis, M.L.; Wolf, J.R.; Heffner, K.L.; Mustian, K.M.; Heckler, C.E.; Peppone, L.J.; Kamen, C.S.; et al. Cognitive Behavioral Therapy for Insomnia Reduces Depression in Cancer Survivors. *J Clin Sleep Med* **2019**, *15*, 129-137, doi:10.5664/jcsm.7586.
57. Persson Asplund, R.; Dagoo, J.; Fjellstrom, I.; Niemi, L.; Hansson, K.; Zeraati, F.; Ziuzina, M.; Geraedts, A.; Ljotsson, B.; Carlbring, P.; et al. Internet-based stress management for distressed managers: results from a randomised controlled trial. *Occup Environ Med* **2018**, *75*, 105-113, doi:10.1136/oemed-2017-104458.
58. Reid, K.J.; Baron, K.G.; Lu, B.; Naylor, E.; Wolfe, L.; Zee, P.C. Aerobic exercise improves

self-reported sleep and quality of life in older adults with insomnia. *Sleep Med* **2010**, *11*, 934-940, doi:10.1016/j.sleep.2010.04.014.
59. Reilmann, R.; McGarry, A.; Grachev, I.D.; Savola, J.M.; Borowsky, B.; Eyal, E.; Gross, N.; Langbehn, D.; Schubert, R.; Wickenberg, A.T.; et al. Safety and efficacy of pridopidine in patients with Huntington's disease (PRIDE-HD): a phase 2, randomised, placebo-controlled, multicentre, dose-ranging study. *Lancet Neurol* **2019**, *18*, 165-176, doi:10.1016/S1474-4422(18)30391-0.
60. Reynolds, C.F., 3rd; Serody, L.; Okun, M.L.; Hall, M.; Houck, P.R.; Patrick, S.; Maurer, J.; Bensasi, S.; Mazumdar, S.; Bell, B.; et al. Protecting sleep, promoting health in later life: a randomized clinical trial. *Psychosom Med* **2010**, *72*, 178-186, doi:10.1097/PSY.0b013e3181c870a5.
61. Rickardsson, J.; Gentili, C.; Holmstrom, L.; Zetterqvist, V.; Andersson, E.; Persson, J.; Lekander, M.; Ljotsson, B.; Wicksell, R.K. Internet-delivered acceptance and commitment therapy as microlearning for chronic pain: A randomized controlled trial with 1-year follow-up. *Eur J Pain* **2021**, *25*, 1012-1030, doi:10.1002/ejp.1723.
62. Rickels, K.; Montgomery, S.A.; Tourian, K.A.; Guelfi, J.D.; Pitrosky, B.; Padmanabhan, S.K.; Germain, J.M.; Leurent, C.; Brisard, C. Desvenlafaxine for the prevention of relapse in major depressive disorder: results of a randomized trial. *J Clin Psychopharmacol* **2010**, *30*, 18-24, doi:10.1097/JCP.0b013e3181c94c4d.
63. Rinke, A.; Neary, M.P.; Eriksson, J.; Hunger, M.; Doan, T.; Karli, D.; Arnold, R. Health-Related Quality of Life for Long-Acting Octreotide versus Placebo in Patients with Metastatic Midgut Neuroendocrine Tumors in the Phase 3 PROMID Trial. *Neuroendocrinology* **2019**, *109*, 141-151, doi:10.1159/000499469.
64. Ritterband, L.M.; Thorndike, F.P.; Ingersoll, K.S.; Lord, H.R.; Gonder-Frederick, L.; Frederick, C.; Quigg, M.S.; Cohn, W.F.; Morin, C.M. Effect of a Web-Based Cognitive Behavior Therapy for Insomnia Intervention With 1-Year Follow-up: A Randomized Clinical Trial. *JAMA Psychiatry* **2017**, *74*, 68-75, doi:10.1001/jamapsychiatry.2016.3249.
65. Roehrs, T.A.; Roth, T. Hyperarousal in insomnia and hypnotic dose escalation. *Sleep Med* **2016**, *23*, 16-20, doi:10.1016/j.sleep.2016.06.008.
66. Roila, F.; Ruggeri, B.; Ballatori, E.; Del Favero, A.; Tonato, M. Aprepitant versus dexamethasone for preventing chemotherapy-induced delayed emesis in patients with breast cancer: a randomized double-blind study. *J Clin Oncol* **2014**, *32*, 101-106, doi:10.1200/JCO.2013.51.4547.
67. Roth, T.; Lines, C.; Vandormael, K.; Ceesay, P.; Anderson, D.; Snavely, D. Effect of gaboxadol on patient-reported measures of sleep and waking function in patients with Primary Insomnia: results from two randomized, controlled, 3-month studies. *J Clin Sleep Med* **2010**, *6*, 30-39.
68. Sadler, P.; McLaren, S.; Klein, B.; Harvey, J.; Jenkins, M. Cognitive behavior therapy for older adults with insomnia and depression: a randomized controlled trial in community mental health services. *Sleep* **2018**, *41*, doi:10.1093/sleep/zsy104.
69. Sakon, M.; Nakamura, M. Darexaban (YM150) prevents venous thromboembolism in Japanese patients undergoing major abdominal surgery: Phase III randomized, mechanical

prophylaxis-controlled, open-label study. *Thromb Res* **2012**, *130*, e52-59, doi:10.1016/j.thromres.2012.06.009.
70. Sauter, C.; Kowalski, J.T.; Stein, M.; Rottger, S.; Danker-Hopfe, H. Effects of a Workplace-Based Sleep Health Program on Sleep in Members of the German Armed Forces. *J Clin Sleep Med* **2019**, *15*, 417-429, doi:10.5664/jcsm.7666.
71. Savard, J.; Savard, M.H.; Ivers, H. Moderators of Treatment Effects of a Video-Based Cognitive-Behavioral Therapy for Insomnia Comorbid With Cancer. *Behav Sleep Med* **2018**, *16*, 294-309, doi:10.1080/15402002.2016.1210148.
72. Schadendorf, D.; Amonkar, M.M.; Milhem, M.; Grotzinger, K.; Demidov, L.V.; Rutkowski, P.; Garbe, C.; Dummer, R.; Hassel, J.C.; Wolter, P.; et al. Functional and symptom impact of trametinib versus chemotherapy in BRAF V600E advanced or metastatic melanoma: quality-of-life analyses of the METRIC study. *Ann Oncol* **2014**, *25*, 700-706, doi:10.1093/annonc/mdt580.
73. Selinheimo, S.; Vuokko, A.; Hublin, C.; Jarnefelt, H.; Karvala, K.; Sainio, M.; Suojalehto, H.; Suvisaari, J.; Paunio, T. Health-related quality among life of employees with persistent nonspecific indoor-air-associated health complaints. *J Psychosom Res* **2019**, *122*, 112-120, doi:10.1016/j.jpsychores.2019.03.181.
74. Sheaves, B.; Freeman, D.; Isham, L.; McInerney, J.; Nickless, A.; Yu, L.M.; Rek, S.; Bradley, J.; Reeve, S.; Attard, C.; et al. Stabilising sleep for patients admitted at acute crisis to a psychiatric hospital (OWLS): an assessor-blind pilot randomised controlled trial. *Psychol Med* **2018**, *48*, 1694-1704, doi:10.1017/S0033291717003191.
75. Si, X.; Zhang, L.; Wang, H.; Zhang, X.; Wang, M.; Han, B.; Li, K.; Wang, Q.; Shi, J.; Wang, Z.; et al. Quality of life results from a randomized, double-blinded, placebo-controlled, multi-center phase III trial of anlotinib in patients with advanced non-small cell lung cancer. *Lung Cancer* **2018**, *122*, 32-37, doi:10.1016/j.lungcan.2018.05.013.
76. Siebmanns, S.; Johansson, P.; Ulander, M.; Johansson, L.; Andersson, G.; Brostrom, A. The effect of nurse-led Internet-based cognitive behavioural therapy for insomnia on patients with cardiovascular disease: A randomized controlled trial with 6-month follow-up. *Nurs Open* **2021**, *8*, 1755-1768, doi:10.1002/nop2.817.
77. Song, Y.; Kelly, M.R.; Fung, C.H.; Dzierzewski, J.M.; Grinberg, A.M.; Mitchell, M.N.; Josephson, K.; Martin, J.L.; Alessi, C.A. Change in Dysfunctional Sleep-Related Beliefs is Associated with Changes in Sleep and Other Health Outcomes Among Older Veterans With Insomnia: Findings From a Randomized Controlled Trial. *Ann Behav Med* **2022**, *56*, 35-49, doi:10.1093/abm/kaab030.
78. Sun, L.; Wang, J.; Shao, L.; Yuan, C.; Zhao, H.; Li, D.; Wang, Z.; Han, P.; Yu, Y.; Xu, M.; et al. Dexamethasone plus oseltamivir versus dexamethasone in treatment-naive primary immune thrombocytopenia: a multicentre, randomised, open-label, phase 2 trial. *Lancet Haematol* **2021**, *8*, e289-e298, doi:10.1016/S2352-3026(21)00030-2.
79. Sweetman, A.; Lack, L.; McEvoy, R.D.; Catcheside, P.G.; Antic, N.A.; Chai-Coetzer, C.L.; Douglas, J.; O'Grady, A.; Dunn, N.; Robinson, J.; et al. Effect of depression, anxiety, and stress symptoms on response to cognitive behavioral therapy for insomnia in patients with comorbid insomnia and sleep apnea: a randomized controlled trial. *J Clin Sleep Med* **2021**,

17, 545-554, doi:10.5664/jcsm.8944.
80. Thiart, H.; Ebert, D.D.; Lehr, D.; Nobis, S.; Buntrock, C.; Berking, M.; Smit, F.; Riper, H. Internet-Based Cognitive Behavioral Therapy for Insomnia: A Health Economic Evaluation. *Sleep* **2016**, *39*, 1769-1778, doi:10.5665/sleep.6152.
81. Thiart, H.; Lehr, D.; Ebert, D.D.; Berking, M.; Riper, H. Log in and breathe out: internet-based recovery training for sleepless employees with work-related strain - results of a randomized controlled trial. *Scand J Work Environ Health* **2015**, *41*, 164-174, doi:10.5271/sjweh.3478.
82. Thorndike, F.P.; Ritterband, L.M.; Gonder-Frederick, L.A.; Lord, H.R.; Ingersoll, K.S.; Morin, C.M. A randomized controlled trial of an internet intervention for adults with insomnia: effects on comorbid psychological and fatigue symptoms. *J Clin Psychol* **2013**, *69*, 1078-1093, doi:10.1002/jclp.22032.
83. Verplaetse, T.L.; Pittman, B.P.; Shi, J.M.; Tetrault, J.M.; Coppola, S.; McKee, S.A. Effect of Lowering the Dose of Varenicline on Alcohol Self-administration in Drinkers With Alcohol Use Disorders. *J Addict Med* **2016**, *10*, 166-173, doi:10.1097/ADM.0000000000000208.
84. Wade, A.G.; Ford, I.; Crawford, G.; McConnachie, A.; Nir, T.; Laudon, M.; Zisapel, N. Nightly treatment of primary insomnia with prolonged release melatonin for 6 months: a randomized placebo controlled trial on age and endogenous melatonin as predictors of efficacy and safety. *BMC Med* **2010**, *8*, 51, doi:10.1186/1741-7015-8-51.
85. Walsh, J.K.; Salkeld, L.; Knowles, L.J.; Tasker, T.; Hunneyball, I.M. Treatment of elderly primary insomnia patients with EVT 201 improves sleep initiation, sleep maintenance, and daytime sleepiness. *Sleep Med* **2010**, *11*, 23-30, doi:10.1016/j.sleep.2009.07.012.
86. Wang, D.; Li, W.; Xiao, Y.; He, W.; Wei, W.; Yang, L.; Yu, J.; Song, F.; Wang, Z. Tryptophan for the sleeping disorder and mental symptom of new-type drug dependence: A randomized, double-blind, placebo-controlled trial. *Medicine (Baltimore)* **2016**, *95*, e4135, doi:10.1097/MD.0000000000004135.
87. Wang, W.; Sawada, M.; Noriyama, Y.; Arita, K.; Ota, T.; Sadamatsu, M.; Kiyotou, R.; Hirai, M.; Kishimoto, T. Tai Chi exercise versus rehabilitation for the elderly with cerebral vascular disorder: a single-blinded randomized controlled trial. *Psychogeriatrics* **2010**, *10*, 160-166, doi:10.1111/j.1479-8301.2010.00334.x.
88. Wang-Weigand, S.; Watissee, M.; Roth, T. Use of a post-sleep questionnaire-interactive voice response system (PSQ-IVRS) to evaluate the subjective sleep effects of ramelteon in adults with chronic insomnia. *Sleep Med* **2011**, *12*, 920-923, doi:10.1016/j.sleep.2011.06.008.
89. Watanabe, N.; Furukawa, T.A.; Shimodera, S.; Morokuma, I.; Katsuki, F.; Fujita, H.; Sasaki, M.; Kawamura, C.; Perlis, M.L. Brief behavioral therapy for refractory insomnia in residual depression: an assessor-blind, randomized controlled trial. *J Clin Psychiatry* **2011**, *72*, 1651-1658, doi:10.4088/JCP.10m06130gry.
90. Watanabe, N.; Horikoshi, M.; Shinmei, I.; Oe, Y.; Narisawa, T.; Kumachi, M.; Matsuoka, Y.; Hamazaki, K.; Furukawa, T.A. Brief mindfulness-based stress management program for a better mental state in working populations - Happy Nurse Project: A randomized controlled trial(). *J Affect Disord* **2019**, *251*, 186-194, doi:10.1016/j.jad.2019.03.067.

91. Weise, C.; Kleinstauber, M.; Andersson, G. Internet-Delivered Cognitive-Behavior Therapy for Tinnitus: A Randomized Controlled Trial. *Psychosom Med* **2016**, *78*, 501-510, doi:10.1097/PSY.0000000000000310.
92. Wigal, S.B.; Childress, A.C.; Belden, H.W.; Berry, S.A. NWP06, an extended-release oral suspension of methylphenidate, improved attention-deficit/hyperactivity disorder symptoms compared with placebo in a laboratory classroom study. *J Child Adolesc Psychopharmacol* **2013**, *23*, 3-10, doi:10.1089/cap.2012.0073.
93. Wigal, T.; Brams, M.; Frick, G.; Yan, B.; Madhoo, M. A randomized, double-blind study of SHP465 mixed amphetamine salts extended-release in adults with ADHD using a simulated adult workplace design. *Postgrad Med* **2018**, *130*, 481-493, doi:10.1080/00325481.2018.1481712.
94. Wigal, T.; Brams, M.; Gasior, M.; Gao, J.; Giblin, J. Effect size of lisdexamfetamine dimesylate in adults with attention-deficit/hyperactivity disorder. *Postgrad Med* **2011**, *123*, 169-176, doi:10.3810/pgm.2011.03.2275.
95. Wiklund, T.; Linton, S.J.; Alfoldi, P.; Gerdle, B. Is sleep disturbance in patients with chronic pain affected by physical exercise or ACT-based stress management? - A randomized controlled study. *BMC Musculoskelet Disord* **2018**, *19*, 111, doi:10.1186/s12891-018-2020-z.
96. Wong, K.Y.; Chung, K.F.; Au, C.H. Low-Intensity Cognitive Behavioral Therapy for Insomnia as the Entry of the Stepped-Care Model in the Community: A Randomized Controlled Trial. *Behav Sleep Med* **2021**, *19*, 378-394, doi:10.1080/15402002.2020.1764000.
97. Yeung, W.F.; Lai, A.Y.; Ho, F.Y.; Suen, L.K.; Chung, K.F.; Ho, J.Y.; Ho, L.M.; Yu, B.Y.; Chan, L.Y.; Lam, T.H. Effects of Zero-time Exercise on inactive adults with insomnia disorder: a pilot randomized controlled trial. *Sleep Med* **2018**, *52*, 118-127, doi:10.1016/j.sleep.2018.07.025.
98. Younossi, Z.M.; Stepanova, M.; Charlton, M.; Curry, M.P.; O'Leary, J.G.; Brown, R.S.; Hunt, S. Patient-reported outcomes with sofosbuvir and velpatasvir with or without ribavirin for hepatitis C virus-related decompensated cirrhosis: an exploratory analysis from the randomised, open-label ASTRAL-4 phase 3 trial. *Lancet Gastroenterol Hepatol* **2016**, *1*, 122-132, doi:10.1016/S2468-1253(16)30009-7.
99. Yu, Z.H.; Xu, X.H.; Wang, S.D.; Song, M.F.; Liu, Y.; Yin, Y.; Mao, H.J.; Tang, G.Z. Effect and safety of paroxetine combined with zolpidem in treatment of primary insomnia. *Sleep Breath* **2017**, *21*, 191-195, doi:10.1007/s11325-017-1462-0.
100. Zhang, J.; Qin, Z.; So, T.H.; Chen, H.; Lam, W.L.; Yam, L.L.; Yan Chan, P.; Lao, L.; Zhang, Z.J. Electroacupuncture Plus Auricular Acupressure for Chemotherapy-Associated Insomnia in Breast Cancer Patients: A Pilot Randomized Controlled Trial. *Integr Cancer Ther* **2021**, *20*, 15347354211019103, doi:10.1177/15347354211019103.

Text S3. The reference list for the included systematic reviews

1. Ahmed, H.; Abushouk, A.I.; Menshawy, A.; Mohamed, A.; Negida, A.; Loutfy, S.A.; Abdel-Daim, M.M. Safety and Efficacy of Ombitasvir/Paritaprevir/Ritonavir and Dasabuvir with or without Ribavirin for Treatment of Hepatitis C Virus Genotype 1: A Systematic Review and Meta-analysis. *Clin Drug Investig* **2017**, *37*, 1009-1023, doi:10.1007/s40261-017-0565-5.
2. Alessi, C.; Vitiello, M.V. Insomnia (primary) in older people. *BMJ Clin Evid* **2011**, *2011*.
3. Bagherzadeh-Azbari, S.; Khazaie, H.; Zarei, M.; Spiegelhalder, K.; Walter, M.; Leerssen, J.; Van Someren, E.J.W.; Sepehry, A.A.; Tahmasian, M. Neuroimaging insights into the link between depression and Insomnia: A systematic review. *J Affect Disord* **2019**, *258*, 133-143, doi:10.1016/j.jad.2019.07.089.
4. Bekele, F.; Hajure, M. Magnitude and determinants of the psychological impact of COVID-19 among health care workers: A systematic review. *SAGE Open Med* **2021**, *9*, 20503121211012512, doi:10.1177/20503121211012512.
5. Bhagavan, C.; Kung, S.; Doppen, M.; John, M.; Vakalalabure, I.; Oldfield, K.; Braithwaite, I.; Newton-Howes, G. Cannabinoids in the Treatment of Insomnia Disorder: A Systematic Review and Meta-Analysis. *CNS Drugs* **2020**, *34*, 1217-1228, doi:10.1007/s40263-020-00773-x.
6. Birling, Y.; Jia, M.; Li, G.; Sarris, J.; Bensoussan, A.; Zhu, X. Zao Ren An Shen for insomnia: a systematic review with meta-analysis. *Sleep Med* **2020**, *69*, 41-50, doi:10.1016/j.sleep.2019.12.023.
7. Campbell, R.; Chabot, I.; Rousseau, B.; Bridge, D.; Nicol, G.; Meier, G. Understanding the unmet needs in insomnia treatment: a systematic literature review of real-world evidence. *Int J Neurosci* **2021**, 1-15, doi:10.1080/00207454.2021.1995383.
8. Carton, L.; Cottencin, O.; Lapeyre-Mestre, M.; Geoffroy, P.A.; Favre, J.; Simon, N.; Bordet, R.; Rolland, B. Off-Label Prescribing of Antipsychotics in Adults, Children and Elderly Individuals: A Systematic Review of Recent Prescription Trends. *Curr Pharm Des* **2015**, *21*, 3280-3297, doi:10.2174/1381612821666150619092903.
9. Chang, P.H.; Chiang, C.H.; Ho, W.C.; Wu, P.Z.; Tsai, J.S.; Guo, F.R. Combination therapy of varenicline with nicotine replacement therapy is better than varenicline alone: a systematic review and meta-analysis of randomized controlled trials. *BMC Public Health* **2015**, *15*, 689, doi:10.1186/s12889-015-2055-0.
10. Chapman, J.L.; Comas, M.; Hoyos, C.M.; Bartlett, D.J.; Grunstein, R.R.; Gordon, C.J. Is Metabolic Rate Increased in Insomnia Disorder? A Systematic Review. *Front Endocrinol (Lausanne)* **2018**, *9*, 374, doi:10.3389/fendo.2018.00374.
11. Chong, J.; Leung, B.; Poole, P. Phosphodiesterase 4 inhibitors for chronic obstructive pulmonary disease. *Cochrane Database Syst Rev* **2017**, *9*, CD002309, doi:10.1002/14651858.CD002309.pub5.
12. Chowdhury, A.I.; Ghosh, S.; Hasan, M.F.; Khandakar, K.A.S.; Azad, F. Prevalence of insomnia among university students in South Asian Region: a systematic review of studies. *J Prev Med Hyg* **2020**, *61*, E525-E529, doi:10.15167/2421-4248/jpmh2020.61.4.1634.
13. da Silva Neto, R.M.; Benjamim, C.J.R.; de Medeiros Carvalho, P.M.; Neto, M.L.R. Psychological effects caused by the COVID-19 pandemic in health professionals: A systematic review with meta-analysis. *Prog Neuropsychopharmacol Biol Psychiatry* **2021**,

104, 110062, doi:10.1016/j.pnpbp.2020.110062.
14. Della Monica, A.; Ferrara, P.; Dal Mas, F.; Cobianchi, L.; Scannapieco, F.; Ruta, F. The impact of Covid-19 healthcare emergency on the psychological well-being of health professionals: a review of literature. *Ann Ig* **2022**, *34*, 27-44, doi:10.7416/ai.2021.2445.
15. Demissie, D.B.; Bitew, Z.W. Mental health effect of COVID-19 pandemic among women who are pregnant and/or lactating: A systematic review and meta-analysis. *SAGE Open Med* **2021**, *9*, 20503121211026195, doi:10.1177/20503121211026195.
16. Dong, F.; Liu, H.L.; Dai, N.; Yang, M.; Liu, J.P. A living systematic review of the psychological problems in people suffering from COVID-19. *J Affect Disord* **2021**, *292*, 172-188, doi:10.1016/j.jad.2021.05.060.
17. Edinger, J.D.; Arnedt, J.T.; Bertisch, S.M.; Carney, C.E.; Harrington, J.J.; Lichstein, K.L.; Sateia, M.J.; Troxel, W.M.; Zhou, E.S.; Kazmi, U.; et al. Behavioral and psychological treatments for chronic insomnia disorder in adults: an American Academy of Sleep Medicine systematic review, meta-analysis, and GRADE assessment. *J Clin Sleep Med* **2021**, *17*, 263-298, doi:10.5664/jcsm.8988.
18. Evans, K.; Rennick-Egglestone, S.; Cox, S.; Kuipers, Y.; Spiby, H. Remotely Delivered Interventions to Support Women With Symptoms of Anxiety in Pregnancy: Mixed Methods Systematic Review and Meta-analysis. *J Med Internet Res* **2022**, *24*, e28093, doi:10.2196/28093.
19. Feng, G.; Han, M.; Li, X.; Geng, L.; Miao, Y. The Clinical Effectiveness of Cognitive Behavioral Therapy for Patients with Insomnia and Depression: A Systematic Review and Meta-Analysis. *Evid Based Complement Alternat Med* **2020**, *2020*, 8071821, doi:10.1155/2020/8071821.
20. Frass, M.; Strassl, R.P.; Friehs, H.; Mullner, M.; Kundi, M.; Kaye, A.D. Use and acceptance of complementary and alternative medicine among the general population and medical personnel: a systematic review. *Ochsner J* **2012**, *12*, 45-56.
21. Fritz, H.; Flower, G.; Weeks, L.; Cooley, K.; Callachan, M.; McGowan, J.; Skidmore, B.; Kirchner, L.; Seely, D. Intravenous Vitamin C and Cancer: A Systematic Review. *Integr Cancer Ther* **2014**, *13*, 280-300, doi:10.1177/1534735414534463.
22. Fuggle, N.; Curtis, E.; Shaw, S.; Spooner, L.; Bruyere, O.; Ntani, G.; Parsons, C.; Conaghan, P.G.; Corp, N.; Honvo, G.; et al. Safety of Opioids in Osteoarthritis: Outcomes of a Systematic Review and Meta-Analysis. *Drugs Aging* **2019**, *36*, 129-143, doi:10.1007/s40266-019-00666-9.
23. Gartlehner, G.; Nussbaumer, B.; Gaynes, B.N.; Forneris, C.A.; Morgan, L.C.; Kaminski-Hartenthaler, A.; Greenblatt, A.; Wipplinger, J.; Lux, L.J.; Sonis, J.H.; et al. Second-generation antidepressants for preventing seasonal affective disorder in adults. *Cochrane Database Syst Rev* **2015**, CD011268, doi:10.1002/14651858.CD011268.pub2.
24. Gottlieb, J.F.; Benedetti, F.; Geoffroy, P.A.; Henriksen, T.E.G.; Lam, R.W.; Murray, G.; Phelps, J.; Sit, D.; Swartz, H.A.; Crowe, M.; et al. The chronotherapeutic treatment of bipolar disorders: A systematic review and practice recommendations from the ISBD task force on chronotherapy and chronobiology. *Bipolar Disord* **2019**, *21*, 741-773, doi:10.1111/bdi.12847.

25. Gupta, L.; Morgan, K.; Gilchrist, S. Does Elite Sport Degrade Sleep Quality? A Systematic Review. *Sports Med* **2017**, *47*, 1317-1333, doi:10.1007/s40279-016-0650-6.
26. Ho, F.Y.; Chan, C.S.; Lo, W.Y.; Leung, J.C. The effect of self-help cognitive behavioral therapy for insomnia on depressive symptoms: An updated meta-analysis of randomized controlled trials. *J Affect Disord* **2020**, *265*, 287-304, doi:10.1016/j.jad.2020.01.062.
27. Hu, N.; Wang, C.; Liao, Y.; Dai, Q.; Cao, S. Smoking and incidence of insomnia: a systematic review and meta-analysis of cohort studies. *Public Health* **2021**, *198*, 324-331, doi:10.1016/j.puhe.2021.07.012.
28. Hu, S.; Lan, T.; Wang, Y.; Ren, L. Individual Insomnia Symptom and Increased Hazard Risk of Cardiocerebral Vascular Diseases: A Meta-Analysis. *Front Psychiatry* **2021**, *12*, 654719, doi:10.3389/fpsyt.2021.654719.
29. Jackson, C.; Freeman, A.L.J.; Szlamka, Z.; Spiegelhalter, D.J. The adverse effects of bisphosphonates in breast cancer: A systematic review and network meta-analysis. *PLoS One* **2021**, *16*, e0246441, doi:10.1371/journal.pone.0246441.
30. Jafari-Koulaee, A.; Bagheri-Nesami, M. The effect of melatonin on sleep quality and insomnia in patients with cancer: a systematic review study. *Sleep Med* **2021**, *82*, 96-103, doi:10.1016/j.sleep.2021.03.040.
31. Jaffer, K.Y.; Chang, T.; Vanle, B.; Dang, J.; Steiner, A.J.; Loera, N.; Abdelmesseh, M.; Danovitch, I.; Ishak, W.W. Trazodone for Insomnia: A Systematic Review. *Innov Clin Neurosci* **2017**, *14*, 24-34.
32. Ji, X.; Liu, J. Subjective sleep measures for adolescents: a systematic review. *Child Care Health Dev* **2016**, *42*, 825-839, doi:10.1111/cch.12376.
33. Jin, X.; Ruiz Beguerie, J.; Sze, D.M.; Chan, G.C. Ganoderma lucidum (Reishi mushroom) for cancer treatment. *Cochrane Database Syst Rev* **2012**, CD007731, doi:10.1002/14651858.CD007731.pub2.
34. Kim, S.H.; Jeong, J.H.; Lim, J.H.; Kim, B.K. Acupuncture using pattern-identification for the treatment of insomnia disorder: a systematic review and meta-analysis of randomized controlled trials. *Integr Med Res* **2019**, *8*, 216-226, doi:10.1016/j.imr.2019.08.002.
35. Kishi, T.; Iwata, N. NMDA receptor antagonists interventions in schizophrenia: Meta-analysis of randomized, placebo-controlled trials. *J Psychiatr Res* **2013**, *47*, 1143-1149, doi:10.1016/j.jpsychires.2013.04.013.
36. Kishi, T.; Sakuma, K.; Nomura, I.; Matsuda, Y.; Mishima, K.; Iwata, N. Brexpiprazole as Adjunctive Treatment for Major Depressive Disorder Following Treatment Failure With at Least One Antidepressant in the Current Episode: a Systematic Review and Meta-Analysis. *Int J Neuropsychopharmacol* **2019**, *22*, 698-709, doi:10.1093/ijnp/pyz040.
37. Koc, Z.; Kaplan, E.; Tanriverdi, D. The effectiveness of telehealth programs on the mental health of women with breast cancer: A systematic review. *J Telemed Telecare* **2022**, 1357633X211069663, doi:10.1177/1357633X211069663.
38. Koopman, A.D.M.; Beulens, J.W.; Dijkstra, T.; Pouwer, F.; Bremmer, M.A.; van Straten, A.; Rutters, F. Prevalence of Insomnia (Symptoms) in T2D and Association With Metabolic Parameters and Glycemic Control: Meta-Analysis. *J Clin Endocrinol Metab* **2020**, *105*, doi:10.1210/clinem/dgz065.

39. Kuenzig, M.E.; Rezaie, A.; Seow, C.H.; Otley, A.R.; Steinhart, A.H.; Griffiths, A.M.; Kaplan, G.G.; Benchimol, E.I. Budesonide for maintenance of remission in Crohn's disease. *Cochrane Database Syst Rev* **2014**, CD002913, doi:10.1002/14651858.CD002913.pub3.
40. Kwon, C.Y.; Lee, B.; Kim, S.H. Effectiveness and safety of ear acupuncture for trauma-related mental disorders after large-scale disasters: A PRISMA-compliant systematic review. *Medicine (Baltimore)* **2020**, *99*, e19342, doi:10.1097/MD.0000000000019342.
41. Lever, I.; Dyball, D.; Greenberg, N.; Stevelink, S.A.M. Health consequences of bullying in the healthcare workplace: A systematic review. *J Adv Nurs* **2019**, *75*, 3195-3209, doi:10.1111/jan.13986.
42. Lin, F.; Su, Y.; Weng, Y.; Lin, X.; Weng, H.; Cai, G.; Cai, G. The effects of bright light therapy on depression and sleep disturbances in patients with Parkinson's disease: a systematic review and meta-analysis of randomized controlled trials. *Sleep Med* **2021**, *83*, 280-289, doi:10.1016/j.sleep.2021.03.035.
43. Liu, F.G.; Tan, A.H.; Peng, C.Q.; Tan, Y.X.; Yao, M.C. Efficacy and Safety of Scalp Acupuncture for Insomnia: A Systematic Review and Meta-Analysis. *Evid Based Complement Alternat Med* **2021**, *2021*, 6621993, doi:10.1155/2021/6621993.
44. Liu, X.; Lu, W.; Liao, S.; Deng, Z.; Zhang, Z.; Liu, Y.; Lu, W. Efficiency and adverse events of electronic cigarettes: A systematic review and meta-analysis (PRISMA-compliant article). *Medicine (Baltimore)* **2018**, *97*, e0324, doi:10.1097/MD.0000000000010324.
45. Lopez-Soto, P.J.; Fabbian, F.; Cappadona, R.; Zucchi, B.; Manfredini, F.; Garcia-Arcos, A.; Carmona-Torres, J.M.; Manfredini, R.; Rodriguez-Borrego, M.A. Chronotype, nursing activity, and gender: A systematic review. *J Adv Nurs* **2019**, *75*, 734-748, doi:10.1111/jan.13876.
46. Ma, Y.; He, B.; Jiang, M.; Yang, Y.; Wang, C.; Huang, C.; Han, L. Prevalence and risk factors of cancer-related fatigue: A systematic review and meta-analysis. *Int J Nurs Stud* **2020**, *111*, 103707, doi:10.1016/j.ijnurstu.2020.103707.
47. Macedo, C.R.; Macedo, E.C.; Torloni, M.R.; Silva, A.B.; Prado, G.F. Pharmacotherapy for sleep bruxism. *Cochrane Database Syst Rev* **2014**, CD005578, doi:10.1002/14651858.CD005578.pub2.
48. Mason, M.; Cates, C.J.; Smith, I. Effects of opioid, hypnotic and sedating medications on sleep-disordered breathing in adults with obstructive sleep apnoea. *Cochrane Database Syst Rev* **2015**, CD011090, doi:10.1002/14651858.CD011090.pub2.
49. Matsunaga, S.; Fujishiro, H.; Takechi, H. Efficacy and Safety of Cholinesterase Inhibitors for Mild Cognitive Impairment:A Systematic Review and Meta-Analysis. *J Alzheimers Dis* **2019**, *71*, 513-523, doi:10.3233/JAD-190546.
50. McCleery, J.; Cohen, D.A.; Sharpley, A.L. Pharmacotherapies for sleep disturbances in Alzheimer's disease. *Cochrane Database Syst Rev* **2014**, CD009178, doi:10.1002/14651858.CD009178.pub2.
51. Mikocka-Walus, A.; Prady, S.L.; Pollok, J.; Esterman, A.J.; Gordon, A.L.; Knowles, S.; Andrews, J.M. Adjuvant therapy with antidepressants for the management of inflammatory bowel disease. *Cochrane Database Syst Rev* **2019**, *4*, CD012680, doi:10.1002/14651858.CD012680.pub2.

52. Miola, A.; Salvati, B.; Sambataro, F.; Toffanin, T. Aripiprazole for the treatment of delusional disorders: A systematic review. *Gen Hosp Psychiatry* **2020**, *66*, 34-43, doi:10.1016/j.genhosppsych.2020.06.012.
53. Miraj, S.; Alesaeidi, S.; Kiani, S. A systematic review of the relationship between dystemprament (sue Mizaj) and treatments and management of diseases (Ilaj and Eslah-e-Mizaj). *Electron Physician* **2016**, *8*, 3378-3384, doi:10.19082/3378.
54. Monahan, K.; Cuzens-Sutton, J.; Siskind, D.; Kisely, S. Quetiapine withdrawal: A systematic review. *Aust N Z J Psychiatry* **2021**, *55*, 772-783, doi:10.1177/0004867420965693.
55. Najafpour, Z.; Fatemi, A.; Goudarzi, Z.; Goudarzi, R.; Shayanfard, K.; Noorizadeh, F. Cost-effectiveness of neuroimaging technologies in management of psychiatric and insomnia disorders: A meta-analysis and prospective cost analysis. *J Neuroradiol* **2021**, *48*, 348-358, doi:10.1016/j.neurad.2020.12.003.
56. Oduwole, O.; Meremikwu, M.M.; Oyo-Ita, A.; Udoh, E.E. Honey for acute cough in children. *Cochrane Database Syst Rev* **2012**, CD007094, doi:10.1002/14651858.CD007094.pub3.
57. Oduwole, O.; Udoh, E.E.; Oyo-Ita, A.; Meremikwu, M.M. Honey for acute cough in children. *Cochrane Database Syst Rev* **2018**, *4*, CD007094, doi:10.1002/14651858.CD007094.pub5.
58. Pagkali, A.; Mamais, I.; Michalinos, A.; Agouridis, A.P. Safety Profile of Niraparib as Maintenance Therapy for Ovarian Cancer: A Systematic Review and Meta-Analysis. *Curr Oncol* **2022**, *29*, 321-336, doi:10.3390/curroncol29010029.
59. Pancheri, C.; Verdolini, N.; Pacchiarotti, I.; Samalin, L.; Delle Chiaie, R.; Biondi, M.; Carvalho, A.F.; Valdes, M.; Ritter, P.; Vieta, E.; et al. A systematic review on sleep alterations anticipating the onset of bipolar disorder. *Eur Psychiatry* **2019**, *58*, 45-53, doi:10.1016/j.eurpsy.2019.02.003.
60. Paumgartten, F.J.; Pereira, S.S.; de Oliveira, A.C. Safety and efficacy of fenproporex for obesity treatment: a systematic review. *Rev Saude Publica* **2016**, *50*, 25, doi:10.1590/S1518-8787.2016050006208.
61. Phillips, E.A.; Gordeev, V.S.; Schreyogg, J. Effectiveness of occupational e-mental health interventions: a systematic review and meta-analysis of randomized controlled trials. *Scand J Work Environ Health* **2019**, *45*, 560-576, doi:10.5271/sjweh.3839.
62. Qiu, R.; Zhang, X.; Zhao, C.; Li, M.; Shang, H. Comparison of the efficacy of dispensing granules with traditional decoction: a systematic review and meta-analysis. *Ann Transl Med* **2018**, *6*, 38, doi:10.21037/atm.2017.10.22.
63. Reeve, B.B.; Mitchell, S.A.; Dueck, A.C.; Basch, E.; Cella, D.; Reilly, C.M.; Minasian, L.M.; Denicoff, A.M.; O'Mara, A.M.; Fisch, M.J.; et al. Recommended patient-reported core set of symptoms to measure in adult cancer treatment trials. *J Natl Cancer Inst* **2014**, *106*, doi:10.1093/jnci/dju129.
64. Reeve, S.; Sheaves, B.; Freeman, D. The role of sleep dysfunction in the occurrence of delusions and hallucinations: A systematic review. *Clin Psychol Rev* **2015**, *42*, 96-115, doi:10.1016/j.cpr.2015.09.001.
65. Rodrigues, T.M.; Castro Caldas, A.; Ferreira, J.J. Pharmacological interventions for daytime sleepiness and sleep disorders in Parkinson's disease: Systematic review and meta-analysis. *Parkinsonism Relat Disord* **2016**, *27*, 25-34, doi:10.1016/j.parkreldis.2016.03.002.

66. Rogers, J.P.; Chesney, E.; Oliver, D.; Pollak, T.A.; McGuire, P.; Fusar-Poli, P.; Zandi, M.S.; Lewis, G.; David, A.S. Psychiatric and neuropsychiatric presentations associated with severe coronavirus infections: a systematic review and meta-analysis with comparison to the COVID-19 pandemic. *Lancet Psychiatry* **2020**, *7*, 611-627, doi:10.1016/S2215-0366(20)30203-0.
67. Rogers, M.A.; Lemmen, K.; Kramer, R.; Mann, J.; Chopra, V. Internet-Delivered Health Interventions That Work: Systematic Review of Meta-Analyses and Evaluation of Website Availability. *J Med Internet Res* **2017**, *19*, e90, doi:10.2196/jmir.7111.
68. Rosner, S.; Englbrecht, C.; Wehrle, R.; Hajak, G.; Soyka, M. Eszopiclone for insomnia. *Cochrane Database Syst Rev* **2018**, *10*, CD010703, doi:10.1002/14651858.CD010703.pub2.
69. Salazar de Pablo, G.; Vaquerizo-Serrano, J.; Catalan, A.; Arango, C.; Moreno, C.; Ferre, F.; Shin, J.I.; Sullivan, S.; Brondino, N.; Solmi, M.; et al. Impact of coronavirus syndromes on physical and mental health of health care workers: Systematic review and meta-analysis. *J Affect Disord* **2020**, *275*, 48-57, doi:10.1016/j.jad.2020.06.022.
70. Samara, M.T.; Huhn, M.; Chiocchia, V.; Schneider-Thoma, J.; Wiegand, M.; Salanti, G.; Leucht, S. Efficacy, acceptability, and tolerability of all available treatments for insomnia in the elderly: a systematic review and network meta-analysis. *Acta Psychiatr Scand* **2020**, *142*, 6-17, doi:10.1111/acps.13201.
71. Scharner, V.; Hasieber, L.; Sonnichsen, A.; Mann, E. Efficacy and safety of Z-substances in the management of insomnia in older adults: a systematic review for the development of recommendations to reduce potentially inappropriate prescribing. *BMC Geriatr* **2022**, *22*, 87, doi:10.1186/s12877-022-02757-6.
72. Serralde-Zuniga, A.E.; Gonzalez Garay, A.G.; Rodriguez-Carmona, Y.; Melendez, G. Fluoxetine for adults who are overweight or obese. *Cochrane Database Syst Rev* **2019**, *10*, CD011688, doi:10.1002/14651858.CD011688.pub2.
73. Seyffert, M.; Lagisetty, P.; Landgraf, J.; Chopra, V.; Pfeiffer, P.N.; Conte, M.L.; Rogers, M.A. Internet-Delivered Cognitive Behavioral Therapy to Treat Insomnia: A Systematic Review and Meta-Analysis. *PLoS One* **2016**, *11*, e0149139, doi:10.1371/journal.pone.0149139.
74. Silang, K.A.; Sohal, P.R.; Bright, K.S.; Leason, J.; Roos, L.; Lebel, C.; Giesbrecht, G.F.; Tomfohr-Madsen, L.M. eHealth Interventions for Treatment and Prevention of Depression, Anxiety, and Insomnia During Pregnancy: Systematic Review and Meta-analysis. *JMIR Ment Health* **2022**, *9*, e31116, doi:10.2196/31116.
75. Sirdifield, C.; Chipchase, S.Y.; Owen, S.; Siriwardena, A.N. A Systematic Review and Meta-Synthesis of Patients' Experiences and Perceptions of Seeking and Using Benzodiazepines and Z-Drugs: Towards Safer Prescribing. *Patient* **2017**, *10*, 1-15, doi:10.1007/s40271-016-0182-z.
76. Sofi, F.; Cesari, F.; Casini, A.; Macchi, C.; Abbate, R.; Gensini, G.F. Insomnia and risk of cardiovascular disease: a meta-analysis. *Eur J Prev Cardiol* **2014**, *21*, 57-64, doi:10.1177/2047487312460020.
77. Sun, P.; Wang, M.; Song, T.; Wu, Y.; Luo, J.; Chen, L.; Yan, L. The Psychological Impact of

COVID-19 Pandemic on Health Care Workers: A Systematic Review and Meta-Analysis. *Front Psychol* **2021**, *12*, 626547, doi:10.3389/fpsyg.2021.626547.
78. Swallow, E.; Song, J.; Yuan, Y.; Kalsekar, A.; Kelley, C.; Peeples, M.; Mu, F.; Ackerman, P.; Signorovitch, J. Daclatasvir and Sofosbuvir Versus Sofosbuvir and Ribavirin in Patients with Chronic Hepatitis C Coinfected with HIV: A Matching-adjusted Indirect Comparison. *Clin Ther* **2016**, *38*, 404-412, doi:10.1016/j.clinthera.2015.12.017.
79. Tandon, N.; Yadav, S.S. Safety and clinical effectiveness of Withania Somnifera (Linn.) Dunal root in human ailments. *J Ethnopharmacol* **2020**, *255*, 112768, doi:10.1016/j.jep.2020.112768.
80. Tang, L.; Liu, W.; Yang, Y.; Han, W.; Li, K. Relationship between sleep and cognitive function in patients with heart failure: A systematic review. *J Psychosom Res* **2020**, *130*, 109913, doi:10.1016/j.jpsychores.2019.109913.
81. Tashakori-Miyanroudi, M.; Souresrafil, A.; Hashemi, P.; Jafar Ehsanzadeh, S.; Farrahizadeh, M.; Behroozi, Z. Prevalence of depression, anxiety, and psychological distress in patients with epilepsy during COVID-19: A systematic review. *Epilepsy Behav* **2021**, *125*, 108410, doi:10.1016/j.yebeh.2021.108410.
82. Thakur, B.; Pathak, M. Burden of Predominant Psychological Reactions Among the Healthcare Workers and General Population During COVID-19 Pandemic Phase: A Systematic Review and Meta-Analysis. *Indian J Community Med* **2021**, *46*, 600-605, doi:10.4103/ijcm.IJCM_1007_20.
83. Thaler, K.J.; Morgan, L.C.; Van Noord, M.; Gaynes, B.N.; Hansen, R.A.; Lux, L.J.; Krebs, E.E.; Lohr, K.N.; Gartlehner, G. Comparative effectiveness of second-generation antidepressants for accompanying anxiety, insomnia, and pain in depressed patients: a systematic review. *Depress Anxiety* **2012**, *29*, 495-505, doi:10.1002/da.21951.
84. Thompson, J.; Stansfeld, J.L.; Cooper, R.E.; Morant, N.; Crellin, N.E.; Moncrieff, J. Experiences of taking neuroleptic medication and impacts on symptoms, sense of self and agency: a systematic review and thematic synthesis of qualitative data. *Soc Psychiatry Psychiatr Epidemiol* **2020**, *55*, 151-164, doi:10.1007/s00127-019-01819-2.
85. Tickell-Painter, M.; Maayan, N.; Saunders, R.; Pace, C.; Sinclair, D. Mefloquine for preventing malaria during travel to endemic areas. *Cochrane Database Syst Rev* **2017**, *10*, CD006491, doi:10.1002/14651858.CD006491.pub4.
86. van Nooten, F.; Treur, M.; Pantiri, K.; Stoker, M.; Charokopou, M. Capsaicin 8% Patch Versus Oral Neuropathic Pain Medications for the Treatment of Painful Diabetic Peripheral Neuropathy: A Systematic Literature Review and Network Meta-analysis. *Clin Ther* **2017**, *39*, 787-803 e718, doi:10.1016/j.clinthera.2017.02.010.
87. Wan, M.; Luo, X.; Wang, J.; Mvogo Ndzana, L.B.; Chang, C.; Li, Z.; Zhang, J. The impact on quality of life from informing diagnosis in patients with cancer: a systematic review and meta-analysis. *BMC Cancer* **2020**, *20*, 618, doi:10.1186/s12885-020-07096-6.
88. Wang, W.L.; Chen, K.H.; Pan, Y.C.; Yang, S.N.; Chan, Y.Y. The effect of yoga on sleep quality and insomnia in women with sleep problems: a systematic review and meta-analysis. *BMC Psychiatry* **2020**, *20*, 195, doi:10.1186/s12888-020-02566-4.
89. Wijarnpreecha, K.; Thongprayoon, C.; Panjawatanan, P.; Ungprasert, P. Insomnia and risk

of nonalcoholic fatty liver disease: A systematic review and meta-analysis. *J Postgrad Med* **2017**, *63*, 226-231, doi:10.4103/jpgm.JPGM_140_17.

90. Wong, W.K.; Li, M.Y.; Yung, P.S.; Leong, H.T. The effect of psychological factors on pain, function and quality of life in patients with rotator cuff tendinopathy: A systematic review. *Musculoskelet Sci Pract* **2020**, *47*, 102173, doi:10.1016/j.msksp.2020.102173.
91. Wu, T.; Jia, X.; Shi, H.; Niu, J.; Yin, X.; Xie, J.; Wang, X. Prevalence of mental health problems during the COVID-19 pandemic: A systematic review and meta-analysis. *J Affect Disord* **2021**, *281*, 91-98, doi:10.1016/j.jad.2020.11.117.
92. Xie, C.L.; Gu, Y.; Wang, W.W.; Lu, L.; Fu, D.L.; Liu, A.J.; Li, H.Q.; Li, J.H.; Lin, Y.; Tang, W.J.; et al. Efficacy and safety of Suanzaoren decoction for primary insomnia: a systematic review of randomized controlled trials. *BMC Complement Altern Med* **2013**, *13*, 18, doi:10.1186/1472-6882-13-18.
93. Xie, Y.; Liu, S.; Chen, X.J.; Yu, H.H.; Yang, Y.; Wang, W. Effects of Exercise on Sleep Quality and Insomnia in Adults: A Systematic Review and Meta-Analysis of Randomized Controlled Trials. *Front Psychiatry* **2021**, *12*, 664499, doi:10.3389/fpsyt.2021.664499.
94. Xu, J.; Deng, Q.; Qin, Q.; Vgontzas, A.N.; Basta, M.; Xie, C.; Li, Y. Sleep disorders in Wilson disease: a systematic review and meta-analysis. *J Clin Sleep Med* **2020**, *16*, 219-230, doi:10.5664/jcsm.8170.
95. Xu, W.; Tan, C.C.; Zou, J.J.; Cao, X.P.; Tan, L. Sleep problems and risk of all-cause cognitive decline or dementia: an updated systematic review and meta-analysis. *J Neurol Neurosurg Psychiatry* **2020**, *91*, 236-244, doi:10.1136/jnnp-2019-321896.
96. Yang, X.Q.; Liu, L.; Ming, S.P.; Fang, J.; Wu, D.N. Tian Wang Bu Xin Dan for Insomnia: A Systematic Review of Efficacy and Safety. *Evid Based Complement Alternat Med* **2019**, *2019*, 4260801, doi:10.1155/2019/4260801.
97. Yoo, I.G. The effects of the type of delivery of cognitive-behavioral therapy for healthcare workers: A systematic review. *J Clin Psychol* **2022**, *78*, 149-166, doi:10.1002/jclp.23215.
98. Yuan, L.; Dai, X.; Yang, M.; Cai, Q.; Shao, N. Potential treatment benefits and safety of roflumilast in COPD: a systematic review and meta-analysis. *Int J Chron Obstruct Pulmon Dis* **2016**, *11*, 1477-1483, doi:10.2147/COPD.S106370.
99. Zhang, L.; Li, J.; Zhao, Y.; Su, Y.; Si, T. Critical evaluation of paliperidone in the treatment of schizophrenia in Chinese patients: a systematic literature review. *Neuropsychiatr Dis Treat* **2016**, *12*, 113-131, doi:10.2147/NDT.S64672.
100. Zhang, S.X.; Chen, R.Z.; Xu, W.; Yin, A.; Dong, R.K.; Chen, B.Z.; Delios, A.Y.; Miller, S.; McIntyre, R.S.; Ye, W.; et al. A Systematic Review and Meta-Analysis of Symptoms of Anxiety, Depression, and Insomnia in Spain in the COVID-19 Crisis. *Int J Environ Res Public Health* **2022**, *19*, doi:10.3390/ijerph19021018.
